# Mesoporous Silica Nanoparticles in Chemical Detection: From Small Species to Large Bio-Molecules

**DOI:** 10.3390/s22010261

**Published:** 2021-12-30

**Authors:** Margarita Parra, Salvador Gil, Pablo Gaviña, Ana M. Costero

**Affiliations:** 1 Instituto Interuniversitario de Investigación de Reconocimiento Molecular y Desarrollo Tecnológico (IDM), Universitat Politècnica de València, Universitat de València, Doctor Moliner 50, 46100 Burjassot, Valencia, Spain; margarita.parra@uv.es (M.P.); salvador.gil@uv.es (S.G.); pablo.gavina@uv.es (P.G.); 2 Departamento de Química Orgánica, Universitat de València, Doctor Moliner 50, 46100 Burjassot, Valencia, Spain; 3 CIBER de Bioingeniería, Biometariales y Nanomedicina (CIBER-BBN), 28029 Madrid, Spain

**Keywords:** mesoporous hybrid materials, optical chemosensors, molecular gates, silica nanoparticles

## Abstract

A recompilation of applications of mesoporous silica nanoparticles in sensing from the last five years is presented. Its high potential, especially as hybrid materials combined with organic or bio-molecules, is shown. Adding to the multiplying effect of loading high amounts of the transducer into the pores, the selectivity attained by the interaction of the analyte with the layer decorating the material is described. Examples of the different methodologies are presented.

## 1. Introduction

The development of mesoporous materials has grown exponentially in recent decades, both the study of their properties and synthetic methods for their preparation as well as their multiple applications. In this review, we limited the subject to the last five years since there are several reviews [1,2,3] and books [4] available that collect previous results. Since the 1990s, when mesoporous silica particles were published for the first time [5], many and very interesting applications were developed in different fields such as catalysis [6], energy applications [7,8] or nano-biomedicine, among others. In the latter, they demonstrated satisfactory in-vitro performance, biocompatibility, non-toxicity and in-vivo bioavailability enhancement [9], and they were applied both in controlled drug release [10,11,12] and in diagnostics [13,14]. Another relevant field is its application in sensing. Electrochemical sensors are already reviewed [15,16] and for this reason, in this review, we will focus on the use of mesoporous silica nanoparticles for the optical detection of different chemical species. The revision is limited to systems related to MCM (Mobil Composition of Matter) or SPB (Santa Barbara Amorphous) materials. In addition, nanoparticle size in most of the revised publication is in the range 50–150 nm, even though there are some examples with higher (200–300 nm) or lower (10–50 nm) diameters. Pore size mostly varied from 2 to 3 nm except when large-pore mesoporous silica nanoparticles (LPMSNs) are used that have pore sizes from 5 to 10 nm.

Three different approaches are used in the development of mesoporous silica material-based sensors:

(1) Probes are loaded inside the pores of the material to preserve or modify their optical or binding properties.
(2) Chemosensors or probes are covalently bound to the surface of the material that is acting as a solid support.
(3) Dyes are loaded inside the pores of the nanomaterials, and these pores are closed with molecular gates. These molecular gates retain the cargo inside the pores and are only opened in the presence of the analyte with the concomitant release of the dye.

In the first approach, the sensing compound is loaded inside the pores without any covalent interaction with the material. The confinement of an organic molecule within the pores of mesoporous materials can protect it against both chemical and photochemical decomposition [17,18]. Thus, several organic fluorophores loaded on mesoporous materials show an improvement in their photophysical properties (brightness, quantum yield, photostability, etc.). The observed improvement can be due to the reduction of phenomena such as the formation of non-luminescent aggregates and inner filter effects [19]. In the same sense, the thermal and photochemical stability as well as the mechanical properties of several lantanides complexes are improved when they are loaded into the mesoporous of silica materials [20,21].

**Figure 1:**
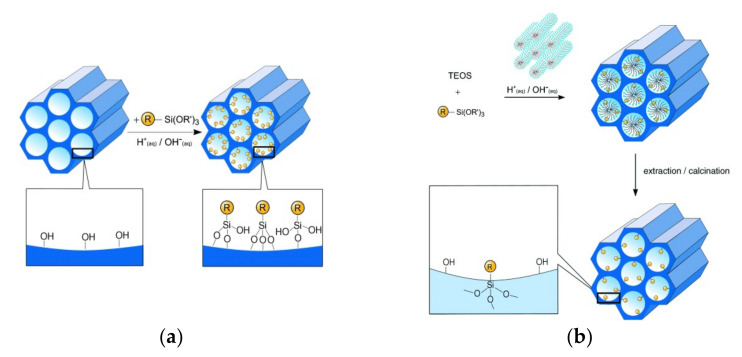
Schematic representation of the different approaches for MCM-41 functionalization: (**a**) post-synthetic functionalization (or grafting); (**b**) direct synthesis (or co-condensation). Reprinted with permission from Frank Hoffmann, Maximilian Cornelius, Jurgen Morell, et al., (2006). Copyright 2006 Wiley-VCH GmbH $ Co. KGaA, Weinheim.

In the second approach, a grafting procedure can be used (Figure 1a). In this case, the functionalization procedure is based on the anchoring of the sensing molecules on the external surface of the mesoporous solid. In this process, a trialkoxysilane (R’-Si (OR)_3_) group bound to the organic molecule reacts with the free silanol groups of the material. The concentration of functional groups from the organic molecule as well as their distribution in the material, is influenced by the reactivity of the trialkoxysilane and by its accessibility to the silanols on the surface of the material. One factor to consider in this type of reaction is the polycondensation of the trialkoxysilane. To prevent the reaction of this precursor with itself, instead of a reaction with the silanols of the material, different parameters should be controlled, such as: solvent, reaction temperature, amount of water adsorbed on the surface and the type of trialkoxysilane [22,23].

Alternatively, co-condensation or direct synthesis (Figure 1b) can be considered. In this method, the surface is modified at the same time the material is synthesized, by including the trialkoxysilane derivative in its structure. By means of this method, it is possible to obtain a great variety of functional groups in the material, as long as these functional groups do not produce a break or deformations in the structure of the mesoporous material. An advantage of this method is that the organic groups are homogeneously distributed throughout the material [24].

**Figure 2:**
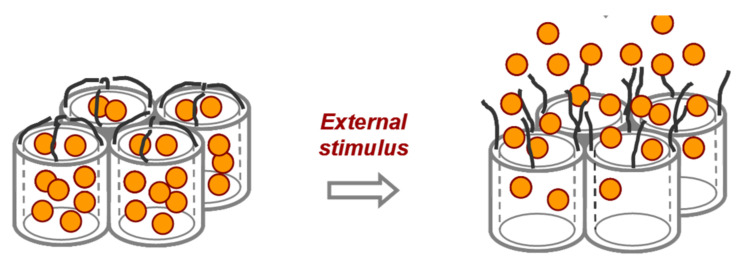
Schematic representation of a mesoporous material capped with molecular gates and open protocol.

Finally, in the third approach, a more sophisticated procedure is used. In this case, the pores are loaded with a dye or fluorophore and capped with a molecule that acts as a molecular gate (Figure 2). This molecular gate presents two different states: closed and open. The change from closed to open must be induced by the presence of the studied analyte in the solution. Thus, in the absence of the analyte, the molecular gate will be closed, and the dye or fluorophore will be confined within the pores, while in the presence of the analyte, the molecular gate will open allowing the dye to escape to the solution, giving rise to the signaling event [25,26,27,28].

Hybrid materials prepared following any of the three described approaches are used for detecting a wide variety of species. In the present review, the studied species are classified in the following groups: cations, anions, radicals, neutral analytes and miscellany.

## 2. Detection Studies

### 2.1. Detection of Cations

Silica nanoparticles are widely used for detecting cations. Several interesting results are summarized in the review published in 2021 for N. Choudhury, B. Saha and P. De [29]. In addition to the interesting references included, there are some recent contributions in this area that will be commented now.

**Figure 3:**
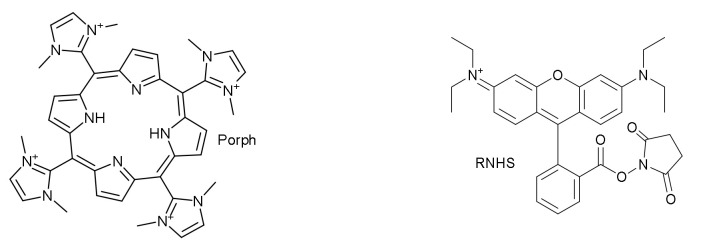
Structure of chemosensors **Porph** and **RNHS**.

Following the first approach indicated in the introduction, E. Oliveira et al. used mesoporous SBA-16 silica nanoparticles. This material consists of a pore size of approximately 5–15 nm and the arrangement of a three-dimensional cubic mesopore corresponding to the space group Im3m [30]. The authors doped this material with a cationic porphyrin bearing imidazolium substituents (**Porph**) or with a rhodamine derivative (**RhNHS**) (Figure 3) [31]. In solution, **Porph** was colorimetrically selective for Cu^2+^, Pb^2+^ and Hg^2+^, the color change being different for each cation. Limits of detection determined for these cations were 5.8, 3.8 and 3.6 ppm for Pb^2+^, Hg^2+^ and Cu^2+^, respectively. By contrast, **RNHS** in acetonitrile did not show any change in the presence of the cations probably because the transduction mechanism seems to need the presence of water in the medium.

When **Porph** and **RNHS** were adsorbed on mesoporous silica SBA-16, two new materials (**SBA-16 @Porph** and **SBA-16@RNHS**) were obtained. At the ppm levels, these new materials did not show important changes in Pb^2+^ and Cu^2+^ sensing when compared with both ligands in the solution. However, a more interesting behavior was observed in the presence of Hg^2+^. Thus, at the ppm level, naked eye observable changes were induced by this cation in aqueous solutions. When similar experiments were carried out at the ppb level of the cations, the changes were not detectable by the naked eye, but they were clearly observed using spectroscopic techniques. In consequence, the new prepared materials gave rise to lower limits of detection than those determined in solution. Additionally, these materials could be used for the removal of Hg^2+^ from aqueous sources.

**Figure 4:**
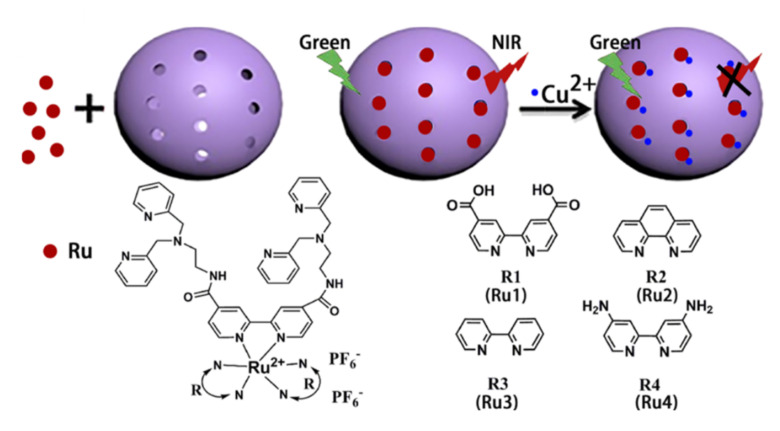
Transduction mechanism in Cu^2+^ detection using a R-LPMSNPs hybrid material. Reprinted with permission from Fangman Chen, Fangnan Xiao, Weibing zhang, et al., (2018). Copyright 2018 ACS Publications.

Following the same approach, Wu et al. [32] used nanomaterials as containers to increase both the sensitivity of Ru-based luminescent sensors as well as the interaction between the Ru-complex and the corresponding analyte. Following previously reported results, the authors used large-pore mesoporous silica nanoparticles (LPMSNs) due to their better mass transfer, diffusivity, and guest penetration when compared with conventional MSNs [33]. The authors reported the synthesis of **NIR Ru-LPMSN** hybrid materials using different Ru-complexes with selective recognition for Cu^2+^. The sensing mechanism is schematized in Figure 4, and it is based on the efficient complexation of Cu^2+^.

Among the different Ru-complexes used, the one containing ligand R4 showed the best results. The quenching of the fluorescence as a function of the Cu^2+^ concentration allowed for the determination of a limit of detection 10.0 × 10^−9^ M. In addition, the sensor was selective to Cu^2+^ in front of other cations. Only Ni^2+^ and Co^2+^ induced a slight quenching of the fluorescence but at a higher concentration (10 μM). The response induced by the cation was reproducible, and the sensor could be regenerated by adding EDTA disodium salt to the material. Finally, the material was able to detect the presence of Cu^2+^ in cells and in vivo imaging in zebrafish larvae. The improvement observed in the system was related to the increased environmental stability of the Ru-complexes that enhances its fluorescence intensity. Additionally, the sensitivity towards Cu^2+^ could be increased through the absorption and accumulation of the cation by the large surface and the big tunnel structure of LPMSNs.

**Figure 5:**
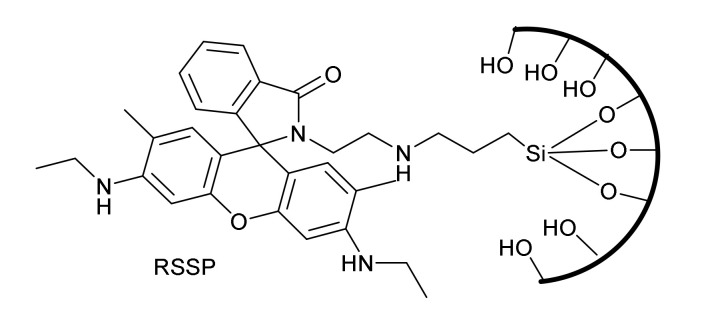
Hybrid material derived from Rhodamine 6G for detecting Fe^3+^.

The second approach for preparing chemosensors involving mesoporous material is related to the covalent binding of a previously synthesized sensor to the corresponding material. Following this approach, Y.-A. Son et al. [34] reported a hybrid material **RSSP** (Figure 5) constituted by a rhodamine 6G derivative grafted to mesoporous silica nanoparticles.

The optical response of this material toward a large number of metal cations (Ag^+^, Na^+^, Li^+^, K^+^, Cs^+^, Hg^2+^, Cu^2+^, Ca^2+^, Cd^2+^, Co^2+^, Fe^2+^, Mg^2+^, Ni^2+^, Pb^2+^, Zn^2+^, Fe^3+^ and Al^3+^) in water was studied. The obtained results showed that the material worked as an Off-On sensor in presence of Fe^3+^. In fact, a strong enhancement of the fluorescence was observed in the presence of the cation (emission at 552 nm upon excitation at 510 nm was enhanced 80-fold in the presence of Fe^3+^ ions) induced by the spirolactame ring opening. Some enhancement of the fluorescence was also observed in the presence of Al^3+^ and Hg^2+^ but at a lower extent. The sensor worked in a reversible way through the addition of EDTA that was able to remove the cation regenerating the initial structure of the binding moiety. The application of the prepared material was evaluated in HeLa cells with positive results as well as in microelectronic devices to generate lab-on-a-chip systems.

Moreover, for detecting Fe^3+^, Gai, Akhtar et al. [35] described the preparation of large pore silica nanoparticles (LPMSNs). The authors studied different synthetic conditions to obtain several materials with different characteristics by using mixed anionic surfactants, sodium dodecyl benzene sulfonate (SDBS) and sodium dodecyl sulphate (SDS) as a template (Table 1).

**MSN1** were non uniform and showed different particle morphologies. The dispersity of nanoparticles was improved in **MSN2**, but they were frangible. The particle size of **MSN-3** ranges from 30 nm to 100 nm with larger aggregates. However, by increasing the amount of APTS, **MSN4** was obtained with similar pore structures to **MSN-3** but in a uniform and monodisperse structure. Finally, changes in temperature (**MSN5**) gave rise to particle aggregation and different particle and pores’ morphologies. Functionalization on the **MSN4** surface by generating the corresponding Schiff base formed between the amino groups of APTES and *N,N*-diphenyl-*N*-(4-formylfenyl)amine gave rise to a material able to detect Fe^3+^ through fluorescent modifications. In fact, the intensity of the sensor was gradually quenched when the cation concentration increased, which could be ascribed to a PET (photoinduced energy transfer) mechanism or a paramagnetic quenching effect of Fe^3+^. Selectivity was tested in front other cations (as Fe^2+^, Cu^2+^, Na^+^, Zn^2+^, Cr^3+^, Co^2+^, Ni^2+^, Mn^2+^ and Mg^2+^) with good results.

**Figure 6:**
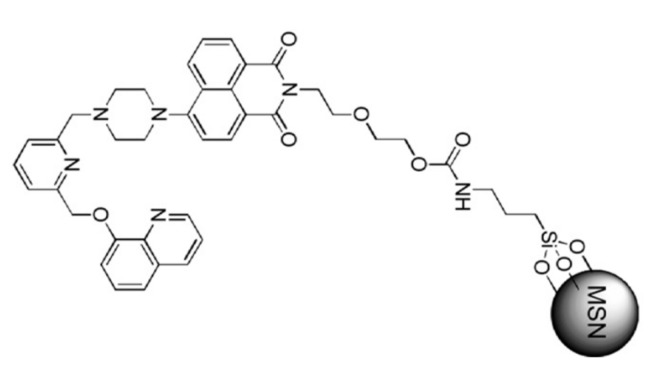
MSN functionalized **FQ** for detection of Ag^+^.

Following the same approach, Zhu et al. [36] prepared a sensing material **FQ** for detecting Ag^+^ with a limit of detection of 7.2 × 10^−6^ M (Figure 6). The fluorescence emission of **FQ** enhanced with the increasing of the Ag^+^ concentration probably due to the formation of a 1:1 complex.

A comparative study between the behavior of the free and the MSN bond receptor demonstrated that both were similar in selectivity. However, the silica material was able to absorb the cation from aqueous solution. In addition, studies carried out for determining the influence of pH showed that the cation could be removed by reducing the pH value. In fact, the use of perchloric acid allows cation liberation, and the solid, after neutralization, could be used again to sense the cation. This process can be repeated at least eight times.

**Figure 7:**
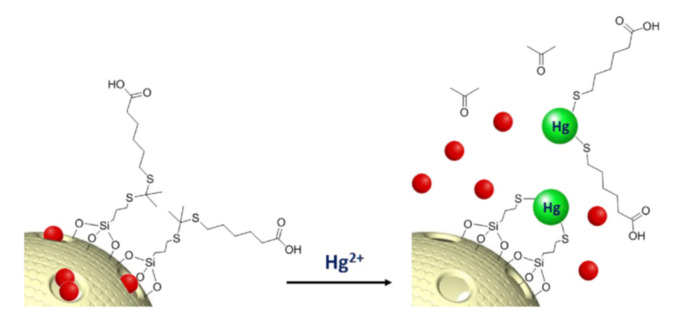
MSN capped with a thioacetal for detecting Hg^2+^. Reprinted with permission from Sandra Jimenez-Falcao, Anabel Villalonga, Jorge Parra-Nieto, Antoni Llopis-Lorente, Paloma Martinez-Ruiz, Ramón Martinez-Mañez, Reynaldo Villalonga (2020). Copyright 2020 Elsevier, Clearance Center.

Some materials are described for detecting cations following the third approach, the use of mesoporous nanoparticles capped with molecular gates. Two of them focus on the sensing of Hg^2+^. In the first one [37], mesoporous silica nanoparticles were loaded with tris(2,20-bipyridyl) dichlororuthenium(II) (Ru(bipy)32þ) as the reporter dye and capped with a thioacetal-containing molecular gate as shown in Figure 7.

In the presence of Hg^2+^ the molecular gate is broken, allowing the release of the dye entrapped into the pores. The dye liberation transmitted the information of the cation presence in solution. The probe showed good selectivity, in the presence of the other metal cation, as only Ag^+^ induced some liberation. In addition, a low limit of detection was determined (60 pM (20 pg/mL)).

**Figure 8:**
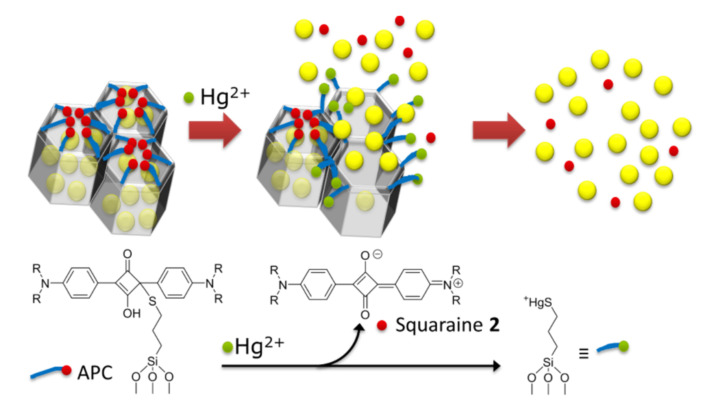
Scheme of analyte-induced indicator release from gated nanoparticles in presence of Hg^2+^. Reprinted with permission from Jérémy Bell, Estela Climent, Mandy Hecht, et al., (2016)**.** Copyright 2016 ACS Publications.

In the second one, mesoporous silica nanoparticles were loaded with a fluorescent BODIPY dye and capped with a squaraine derivative. The pores were subsequently closed with a bulky stopper unit that can be selectively removed through a reaction with the target species Hg^2+^ as shown in Figure 8.

This gated nanomaterial could be incorporated in a biphasic droplet-assisted microfluidic sensor for Hg^2+^ trace detection. The sensor could be built, without sacrificing its efficiency, from simple and commercially available components (conventional optomechanics, PTFE/PFA tubes and a LED). Detection limits of 20 ppt are attained, which are below those required in drinking water in the U.S. and the EU [38].

**Figure 9:**
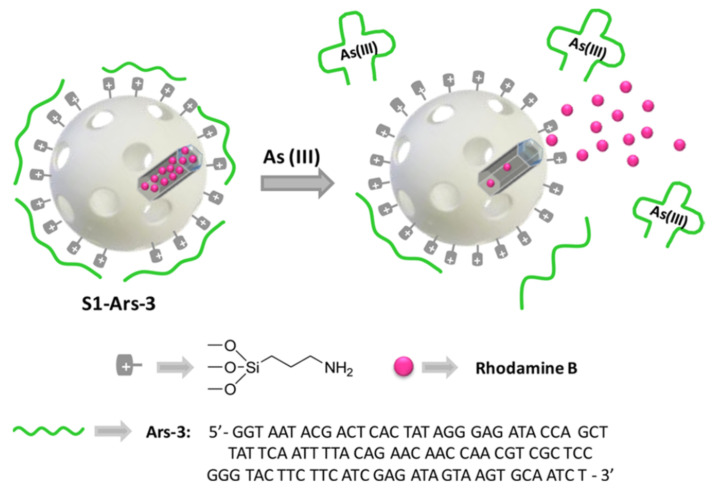
Detection of As^3+^ using mesoporous silica nanoparticles loaded with rhodamine B and capped with an aptamer. Reprinted with permission from Mar Oroval, Carmen Coll, Andrea Bernardos, et al., (2017). Copyright 2017 ACS Publications.

A more elaborated design was used in a probe for detecting As^3+^ using aptamer-capped nanomaterials **S1-ARS-3**. In this occasion [39], the reporter dye was rhodamine B that remained entrapped inside the pores due to the presence of an aptamer (Figure 9).

In the absence of the cation, a poor rhodamine B liberation was observed. By contrast, in the presence of As^3+^, the dye release achieved 80% of the maximum value. On the other hand, the probe showed a clear selectivity when compared with a large number of possible interferents (water containing 50 ppb of As^3+^, As^5+^, Ag^+^, Pb^2+^, K^+^, Na^+^, Fe^3+^, Fe^2+^, Mg^2+^ and Ca^2+^). Based on the release experiments, a limit of detection of 0.9 ppb was determined. The probe was also tested in real samples with recovery levels within the 91−109% range indicating the suitability material for the determination of As^3+^ ions in real environments.

### 2.2. pH Measurements

**Figure 10:**
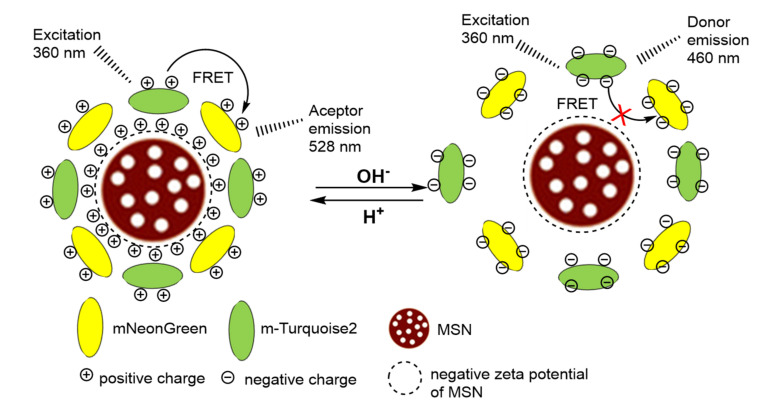
pH sensor based on adsorption of proteins on MSN that regulates the FRET interaction between both proteins.

Related also with the detection of positive charged species, Wang et al. [40] reported a MSN-based probe. Results demonstrated that it showed feasible sensitivity and selectivity for pH measurement in hosting solutions. The sensing mechanism was based on the in situ-assembly induced by the pH of two proteins mTurquoise2 (CFP) and mNeonGreen (YFP) showing type FRET behavior. The adsorption of the proteins on the mesoporous silica nanoparticles was pH dependent, and, in consequence, the FRET effect, that depends on the presence of both proteins in close proximity, could be related with pH as shown in Figure 10. The material showed a good linearity with the pH in the range of 5.5–8.0.

Selectivity was studied using a large number of cations as well as several biomolecules (μM: Mn^2+^, Co^2+^, Ni^2+^, Ba^2+^, Cys (cysteine), GSH (glutathione), Glu (glucose) and H_2_O_2_; 1 mM Mg^2+^, Ca^2+^, SO_4_^2−^ and CO_3_^2−^; 10 mM K^+^, 100 mM Na^+^ and Cl^−^). Only high concentrations of bovine serum albumin (BSA) interfered in the measure. The probe was tested in complexes’ biological samples, and it was demonstrated that it could monitor both proton releasing and proton consuming reactions effectively. Specifically, lipase catalyzed the hydrolysis of ethyl acetate, and urease/urea reactions were tested.

### 2.3. Detection of Radicals

**Figure 11:**
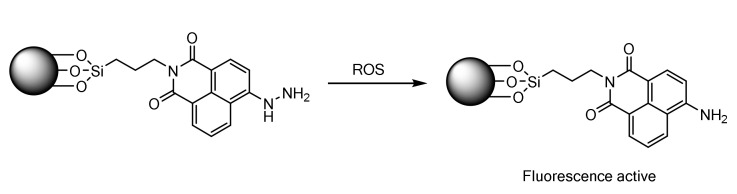
Schematic presentation of N-N bond cleavage of hydrazine group upon attack by ROS.

An important field of study is the detection of reactive oxygen species (ROS), especially in biological systems. In this sense, they have special relevance in cancerous processes and other diseases, since they participate in reactions that can cause cell damage [41]. The development of fluorescent sensors for these species has encountered two obstacles: on the one hand, water-soluble systems are needed, and, on the other, being highly reactive species, the interaction with the sensor normally causes a fluorescence quenching, losing sensitivity. In 2018, a new fluorescent sensor based on the amino and hydrazino derivatives of 1,8-naphthalimide linked to MCM 41 was published, giving rise to a hybrid fluorescent material (Figure 11) [42]. Compared to that of the amine group, the hydrazine group has a relatively less electron donating capability due to the electron withdrawing effect of the second nitrogen atom; the breakdown of the N-N bond by free radicals caused a considerable increase in fluorescence intensity at λ_exc_ = 540 nm, in the acetonitrile:water solvent.

**Figure 12:**
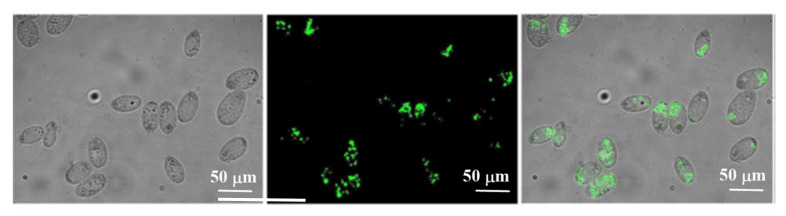
Fluorescence microscopic images of *Tetrahymena* cells incubated with 50 lg/mL for 30 min. Left panel: DIC images, Middle panel: Fluorescence images and Right panel: Merged images. Reprinted with permission from Gaurav Jha, Subhasis Roy, Prabhat Kumar Sahu, Somnath Banerjee, N. Anoop, Abdur Rahaman, Moloy Sarkar (2018). Copyright 2020. Elsevier, Clearance Center.

The authors checked the selectivity towards biologically important cations and anions (Fe^2+^, Fe^3+^, Cu^2+^, Cl^−^, Br^−^, I^−^ NO_3_^−^), and only the free radicals (HO•, t-BuO•, CH_3_•) gave a response. In a study of pH, maximum fluorescence intensity could be obtained in a highly acidic range. Cellular studies with *Tetrahymena* cells suggested that the system was non-toxic and cell permeable, which demonstrated its potential use towards biological applications. Figure 12 shows fluorescence microscopy images of the Tetrahymena cell in the presence and absence of the sensor after a 30 min incubation with the starved cells.

### 2.4. Detection of Anions

**Figure 13:**
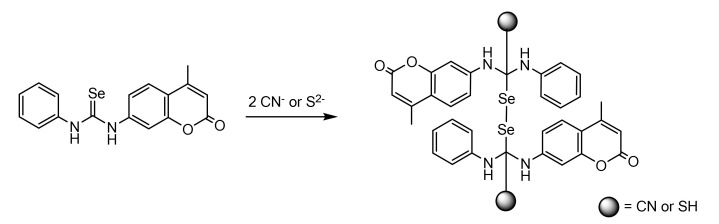
Proposed detection mechanism of L for CN^−^ and S^2−^ in solution.

Based on the first approach described in the introduction, Martínez-Máñez et al. [43] explored the use of selenourea-based anion sensors. In addition to their interesting chemical reactivity, selenoureas exhibit anticancer activity, antioxidant properties, enzyme inhibition and DNA binding properties [44]. The selenourea showed in Figure 13 was a suitable probe for the chromofluorogenic sensing of CN^−^ and S^2−^. In the absorption spectrum, the loss of the bands from the free sensor and the formation of a new absorption band at 353 nm, when any of the anions was present in the medium, were observed. The limit of detection (LOD) was 1.0 × 10^−5^ M and 9.8 × 10^−6^ M for sulphide and cyanide, respectively. Additionally, a partial quenching of the fluorescence emission of the free ligand was observed for the sulphide anion, but no changes were induced by cyanide. The spectroscopy and LR-ESI-MS spectrometry results support a sensing mechanism based on the formation of a diselenide derivative.

The insolubility of the ligand in water precluded its use in this medium. To resolve this problem, the sensor was embedded into functionalized MSNs containing hydrophobic pores. In this case, the system worked as an On–Off fluorescent chemosensor for S^2−^ recognition in pure water, and an enhanced selectivity towards sulphide vs. cyanide was observed.

**Figure 14:**
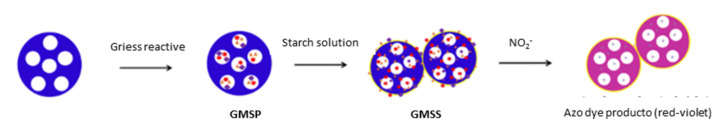
Nitrite detection using the Greiss reagent adsorbed on mesoporous silica nanoparticles. Griess Reagent: sulfanilamide and *N*-1-napthylethylenediamine dihydrochloride under acidic (phosphoric acid) conditions.

Following the same approach, a chromogenic sensor for nitrite was described. Many efforts have been made to detect this analyte as nitrites are important both environmentally and in health. The concentration of nitrites is a measure of the quality of water. Nitrites interfere in the transport of oxygen and promote the production of N-nitrosamines with serious repercussions in organisms [45]. The published sensor was based on the use of the classic Griess reagent which, made up of sulphanilic acid and naphthyleniamine, gives rise to an azo dye in the presence of nitrites [46]. This reagent was loaded on mesoporous silica nanoparticles with both physical and chemical absorption. A complete characterization of the material was described, and in the presence of nitrite a red–violet product occurred immediately (Figure 14). The equilibrium was reached after 3 min with a low detection limit (15.0 μg L^−1^). The application of the optimized method developed in real samples (seawater samples collected in Phuket, Thailand) showed a good correlation with the results obtained from the standard spectrophotometric method.

**Figure 15:**
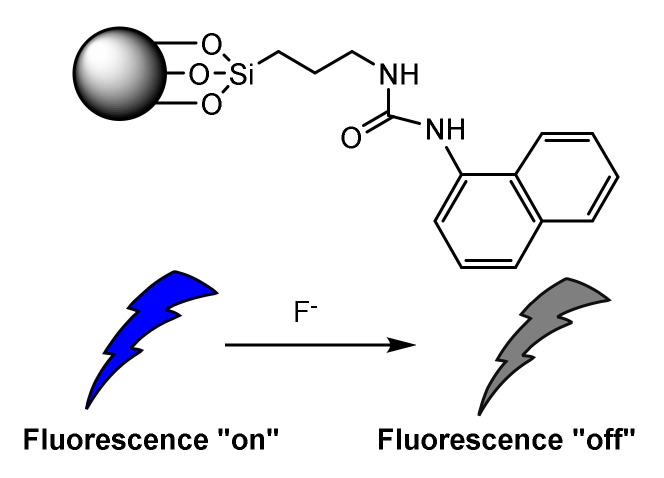
ON-OFF fluorescence detection of Fluoride by a naphthyl-urea.

Following the second approach previously indicated, Wang et al. described a material for detecting fluoride [47]. This anion is one of the most studied, due to its strong tendency to form a hydrogen bond that causes a change in the optical properties of many organic moieties. The system reported by Wang was a novel mesoporous silica nanostructure functionalized with a naphthylurea (Figure 15) that shows a dramatic change in the ultraviolet (UV) absorption spectra and a quenching of the fluorescence emission at 395 nm in the presence of fluoride. A strong dependence of the sensor concentration was observed, and no interferents were detected. The authors indicate that the advantages of this organic-inorganic hybrid system are: higher stability of the molecular-based sensor; less phase separation and leaching of emissive molecules; and the presence of two independent signals (ultraviolet absorption and emission spectra) to express the fluoride recognition process simultaneously.

**Figure 16:**
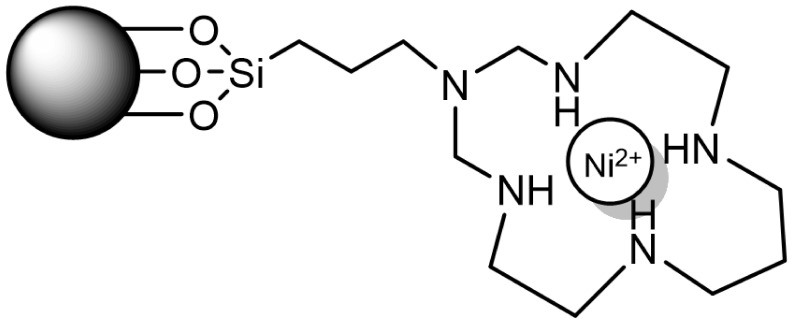
Ni^2+^ complex gated used as cyanide sensor.

Optical detection of cyanide in water has been achieved by using mesoporous silica nanoparticles loaded with [Ru(bipy)_3_]^2+^, functionalized with macrocyclic Ni^2+^ complex subunits (Figure 16), and capped with a sterically hindering anion (hexametaphosphate). Cyanide selectively induces demetallation of Ni^2+^ complexes with the simultaneous removal of the capping anions from the silica surface. This fact allows the release of the dye and the consequent increase in fluorescence intensity. The response is highly selective and sensitive towards cyanide with a limit of detection of 2 μM [48].

Finally, using both approaches simultaneously, a luminescent material for detecting peroxynitrite was reported. Peroxynitrite is a biological oxidant which is involved in several diseases, many of them related to inflammatory processes [49].

**Figure 17:**
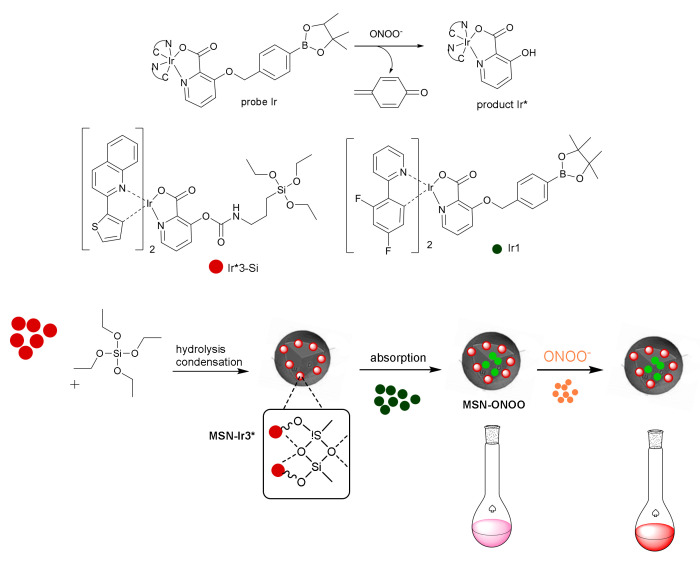
Schematic illustration of sensing mechanism for peroxynitrite.

Various luminescent methods were developed for the detection of peroxynitrite, and one of the problems observed is the interference produced by the endogenous autofluorescence emission of fluorophores [50]. An ultrafast responsive phosphorescent nanohybrid material was developed in 2018 for detecting an elevated peroxynitrite concentration in vitro (RAW 264.7 cells) and endogenous peroxynitrite in vivo (living zebrafish and mouse) with high selectivity [51]. The nanoprobe consisted of mesoporous silica nanoparticles functionalized with an Ir complex (MSN-Ir3*) and embedded with another iridium complex **Ir1** (Figure 17). This material, **MSN-ONOO**, displayed two emission bands, one around 474 nm corresponding to the emission of complex **Ir1** and the other around 605 nm from MSN-Ir3*. The presence of both bands gives rise to a white-pink phosphorescence color. Upon addition of peroxynitrite, the phosphorescence intensity at 474 nm decreases, while the band at 605 nm practically does not change. These two effects give rise to a final red color. **MSN-ONOO** was employed to detect peroxynitrite in vitro (via ratiometric photoluminescence imaging) and in vivo (via time-resolved photoluminescence imaging). Using this photoluminescent nanoprobe, endogenous peroxynitrite was visualized in vivo with a high signal to noise ratio.

### 2.5. Detection of Neutral Analytes

Mesoporous materials are broadly used in the detection of neutral species such as gases, biomolecules, abuse drugs and other organic analytes. The characteristics and moldable size of these materials allow the sensing to be performed in complexes’ matrixes.

#### 2.5.1. Mesoporous Silica Nanoparticles in Gas Sensing

Many gases or gas solutions can be detected or even quantified by using mesoporous materials. Their high surface specific areas are favorable for gas diffusion and adsorption-desorption processes, both essential for gas sensing. Sensing of many gases (sulphur dioxide, hydrogen sulphide, formaldehyde, nitric oxide, warfare gases, volatile organic compounds or oxygen) by means of these materials is recently described.

By using material loaded with an appropriate sensor, Meng et al. detected SO_2_ using a mixture of zinc chloride, sodium nitroprusside and hexamine as an indicator that was loaded into a **SiO_2_@MCM-41**. The resulting white powder turned into red in the presence of SO_2_, with a LOD of 2 ppm [52].

The application of silica nanoparticles in the sensing of oxygen recently attracted attention. It is well known that optical oxygen sensors rely on the fact that oxygen is a powerful quenching agent. In addition, the efficiency of sensing can be improved by assembling the luminescent molecules into the pores of mesoporous silica materials [53]. Among the different dye molecules, the quenching, promoted by molecular oxygen of the phosphorescence of different metal complexes, is mostly used.

In some cases, the metal complex is loaded into the pores. Thus, Zhang et al. [54] encapsulated a [(Ru(dpp)_3_)]Cl_2_ complex into a deformable hollow mesoporous organosilica nanoparticles (HMONs) in order to monitor oxygen, in real-time. HMONs are a type of organic-inorganic hybrid materials that in addition to the advantages of traditional hollow mesoporous silica have different organic functional groups in the mesoporous silica shell. Detection was carried out during photodynamic therapy experiments promoted by a photosensitizer, chlorin e6, that was incorporated along with the ruthenium complex into the 5.4 nm pores of a thioether-bridged deformable HMON. This assembly, able to measure O_2_ concentrations ranging from 1 to 20% in solution, was tested both in vitro and in vivo. A relation between the amount of ROS generated on laser irradiation and oxygen consumption during PDT in real time could be established.

**Figure 18:**
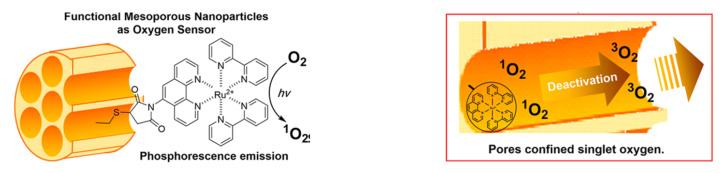
Sensing of oxygen by Ru complex grated on Si-MSM with deactivation of singlet oxygen. Reprinted with permission fromNatsuko Kitajima, Yui Umehara, Aoi Son, et al., (2018). Copyright 2018. ACS Publication.

The application of the former protocol for detecting hypoxic tissues must cope with the drawbacks of the toxicity of the complexes themselves and the generation of an oxygen singlet via an energy transfer from the excited state of the metal. Both problems can be solved by anchoring the ruthenium complex to mesoporous silica material, as demonstrated by Tanabe et al. [55] Inside the 4 nm pores of a thiol-modified MSN, a bipyridine ruthenium complex was anchored through a phenanthroline derivative. The immobilization of the complex into the pores reduces its cytotoxicity while maintaining its phosphorescence properties, allowing a reversible quantification of oxygen. In addition, the singlet oxygen eventually generated is deactivated to the ground state triplet oxygen before leaking from the pore (Figure 18). The authors apply the method both for in vitro, hypoxic HeLa cells and in vivo, ischemia-based hypoxia in one leg.

Following the same approach, Ding et al. attached covalently a pyronine (PYR) derivative to mesoporous MNPs. Pyronine is known to react with hydrogen sulphide, and, in doing so, PYR fluorescence is extinguished. Using the new material, a low detection limit of 103.6 nM to H_2_S was attained [56].

**Figure 19:**
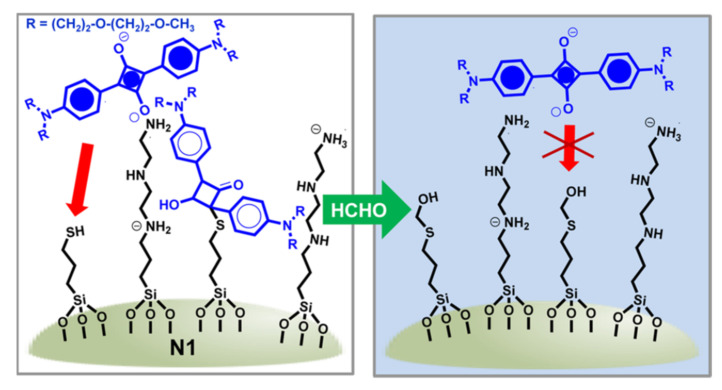
Schematic representation of the colorimetric detection of formaldehyde using polyamines and shorter chain thiols on silica nanoparticles.

Silica nanoparticles could also be used as scaffolds where a layer can be assembled. By combining polyamines and a shorter chain thiol, Martínez-Mañez et al., prepared a hybrid material for selectively detecting formaldehyde [57]. The paradigm, as can be seen in Figure 19, resulted in the loss of coloration of the squaraine blue dye on a reaction with thiols. However, in the presence of formaldehyde, a thio-hemiacetal was formed, preventing its reaction with the squaraine and the blue stain remains. Bulky polyamines generated a highly polar environment around thiols, and only a reaction with the small and polar formaldehyde, but not with longer aldehydes, was favored.

**Figure 20:**
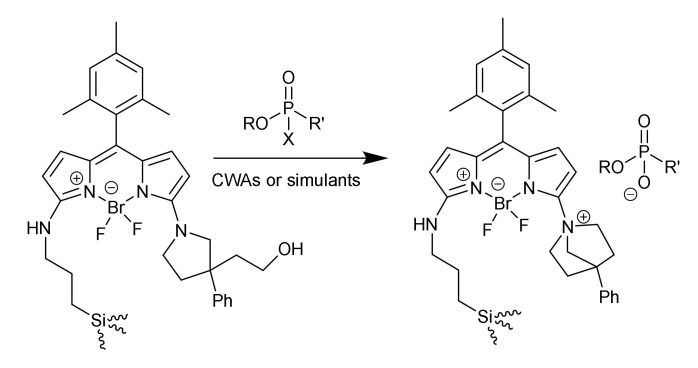
Sensing paradigm for organophosphorus nerve agents and their simulants.

Two works of Rurack et al. for the sensing of nerve agents [58,59] rely on the lack of fluorescence of a 2-amino BODIPY derivative. However, after the reaction with organophosphorus nerve agents, a highly fluorescent bicyclic derivative was generated (Figure 20). Grafting of this dye to mesoporous material allowed the detection to be performed in water, in the pM range, because the material prevents the dye from aggregating. In the same trend, when this type of dye was anchored to pores of mesoporous silica microparticles, higher stability of the material was achieved, and a strip test could be prepared able to detect nervous agents, in the µg m^−3^ range, in a few seconds.

**Figure 21:**
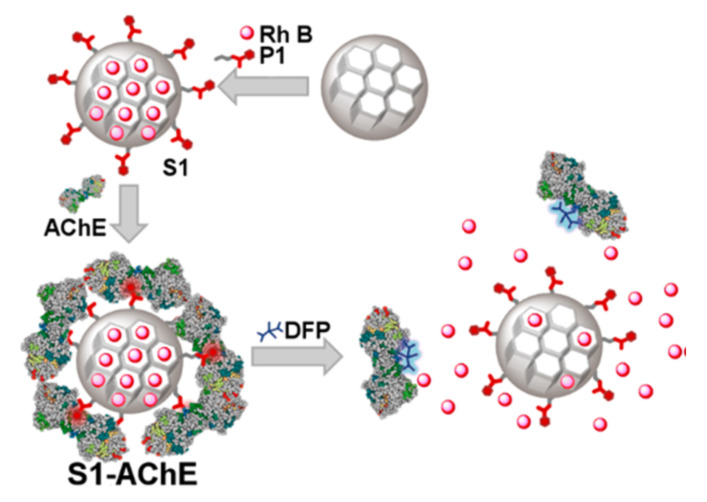
Schematic representation of the acetylcholinesterase (AChE), as molecular gate, on MCM-41 for DFP sensing.

An alternative way to detect organophosphorus nerve agents was developed by Sancenón et al. [60]. In this case, the sensing paradigm was the release of a dye from the pores of gated mesoporous Si-NPs (approach three) on the reaction of nerve agents with acetylcholinesterase (AChE), the same reaction that causes its toxicity. As can be seen in Figure 21, after loading rhodamine B dye into the pores of MCM-41, the surface was grafted with a derivative of pyridostigmine, a well-known AChE inhibitor. After adding AChE, the pores were blocked, entrapping the dye inside. On interaction with a stronger inhibitor, such a nerve agent simulant, the AChE, was removed, and the dye was liberated.

**Figure 22:**
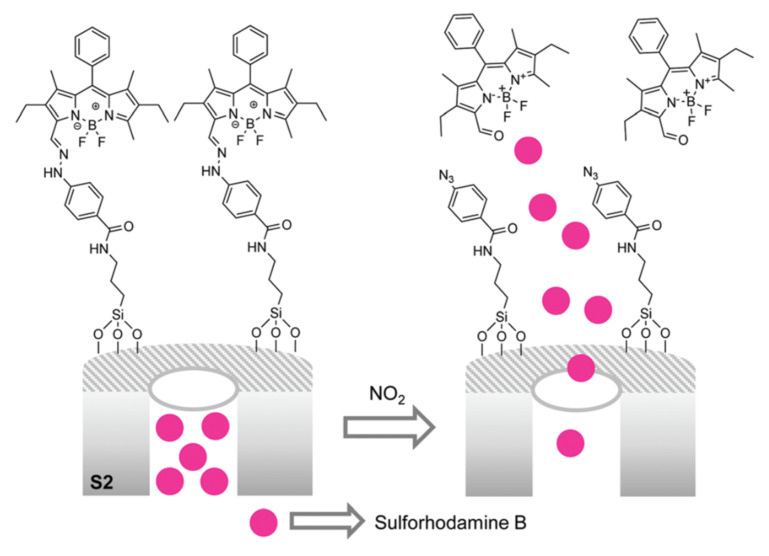
Schematic representation of detection of NO_2_ using the molecular gate-silica nanoparticles loaded with dye system.

The methodology of molecular gates was used by Costero et al. [61] for the detection of NO_2_. The opening of the gate relies on the rupture of an aryl-hydrazone on a reaction with nitric dioxide. Using a difluoroboron-dipyrromethene (BODIPY) derivative hydrazone to block the pores of a MSM-51 loaded with sulforhodamine B, a material able to sense the presence of NO_2_ was prepared. As can be seen in Figure 22, the opening of the pores is concomitant with the liberation of a BODIPY aldehyde dye. However, after a couple of minutes, the multiplying effect of this method becomes apparent, and the color of the sulforhodamine B predominates.

#### 2.5.2. Mesoporous Silica Nanoparticles in the Sensing of Other Neutral Molecules

The immobilization of fluorescent molecules into inorganic matrices renders photofunctional hybrid materials. Thus, grafting on mesoporous materials can promote the fluorescence of those compounds that exhibit aggregation-induced emission (AIEgens), such as tetraphenylethene derivatives, due to the restriction of the intramolecular vibration and rotation in rigid silica networks. Yu et al., prepared this type of material and observed that its fluorescence vanished in the presence of different analytes such as nitrophenols and the antibiotics furazolidone and nitrofurazone. The quenching can be attributed to fluorescence resonance energy transfer from AIEgens to analytes [62].

**Figure 23:**
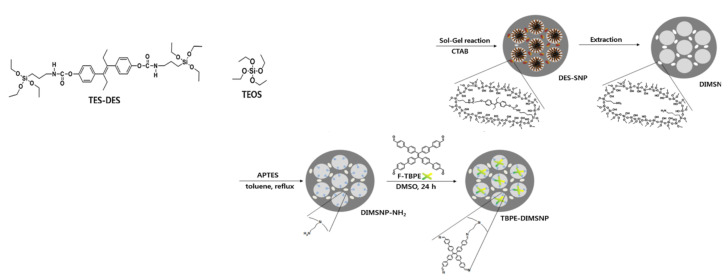
Schematic route of the preparation of TBPE-grafted, DES imprinted mesoporous silica nanoparticles (TBPE-DIMSNPs). Reprinted with permission from Youngdo Kim, Kyoung Min Lee, Ji Young Chang (2017). Copyright 2017. Elsevier, Clearance Center.

In a step further, Chang et al. [63] combined the immobilization of a TPE derivative (tetra(biphenyl-4-yl)ethane, TBPE) and molecular imprinted MSM, for the fluorescent sensing of diethylstilbestrol. In this way, the generated pores of the MSM were pre-arranged, with two binding sites across the pore, to host by a specific binding the target analyte. After that, the TBPE was grafted as a signal transducer. The degree of fluorescence quenching of the material was proportional to the concentration of diethylstilbestrol, and the material could be regenerated after extraction of the entrapped analyte (Figure 23).

**Figure 24:**
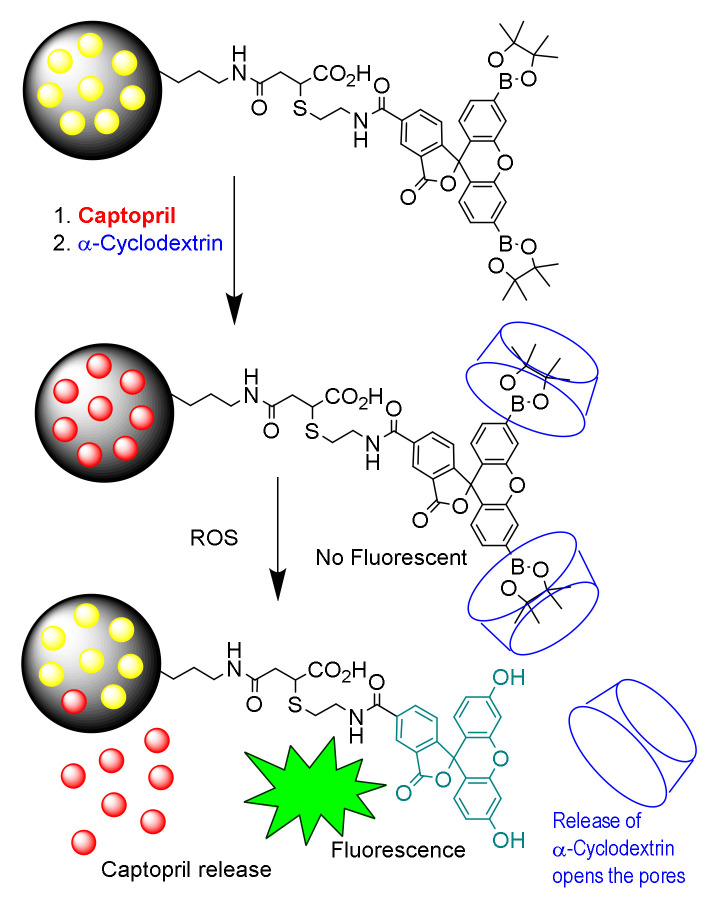
Scheme for the detection of H_2_O_2_ and concomitant treatment of heart failure.

The work of Thao et al. [64] illustrates a combination of sensing and targeted drug release from MSNs by means of molecular gate opening triggered by H_2_O_2_. A non-fluorescent dipinacolboronic-fluoresceine derivative was attached by a 12-atom link to MCM-41. The material was loaded with captopril, a therapeutic drug of heart failure, prior to the formation of an inclusion complex between the dipinacolboronic groups with α-CDs. An increase in ROS closely correlates with an extent of heart failure. The system paradigm is that, in the presence of H_2_O_2_, the boronic groups will be oxidized, rendering fluorescein-MSM with the concomitant loose of the α-CDs. This, in turn, allows the captopril to exit the pores and treat the heart failure (Figure 24). This therapeutic system was illustrated on a KillerRed heart failure transgenic zebrafish model. The material was able not only to sense the oxidative stress-induced heart failure but also to improve the heartbeat rate and cardiac output.

**Figure 25:**
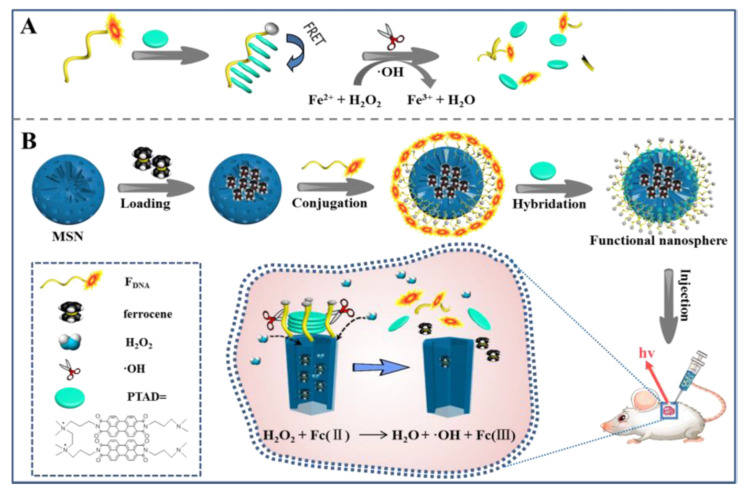
Paradigm of sensing procedure of H_2_O_2_, through a Fenton reaction. (**A**) PTAD-aggregated quenching of FDNA fluorescence and H_2_O_2_-induced fluorescence restoration via Fenton reaction. (**B**) Construction of the activable functional nanosphere by integrating the functional DNA and ferrocene with MSN, and its application in living system for H_2_O_2_ detection. Reprinted with permission from Changhui Liu, Weiju Chen, Zhihe Qing, et al., (2016). Copyright 2016 ACS Publications.

Qing, Wang et al. reported another approach to sense hydrogen peroxide in vivo (Figure 25) through a Fenton reaction in a functional nanosphere, Fc@MSN-FDNA/PTAD. The probe was fabricated from the mesoporous silica nanoparticle (MSN), a Fenton reagent of ferrocene (Fc), ROX-labeled DNA (F_DNA_) and a cationic perylene derivative (PTAD). The ferrocene molecules are loaded inside the pores whereas the external surface is functionalized with F_DNA_. Hybridation with PTAD gives rise to the capped material. The presence of PTDA in the probe simultaneously quenches the ROX emission. H_2_O_2_ can permeate into the nanosphere and react with ferrocene to produce hydroxyl radical (·OH), which cleaves F_DNA_, detaching ROX from PTAD. In this situation, the fluorescence of ROX is recovered. Under physiological conditions, H_2_O_2_ can be determined from 5.0 nM to 1.0 μM with a detection limit of 2.4 nM. In vivo applications were demonstrated for fluorescence imaging of exogenous and endogenous H_2_O_2_ in cells and mice. Compared with other methods, it has the advantage of being rapid, biocompatible and permeable to cell membranes [65].

#### 2.5.3. Mesoporous Silica Nanoparticles in Sensing of Biogenic Analytes

**Figure 26:**
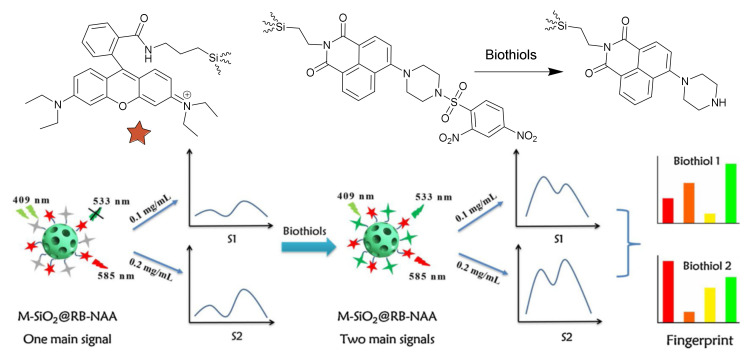
Fluorescence Mini sensor array on Si-MSM for the sensing of biothiols. Reprinted with permission from Zhipeng Gao, Zhaojuan Wang, Min Qiao, Haonan Peng, Liping Ding, Yu Fang (2020). Copyright 2020. Elsevier, Clearance Center.

Silica mesoporous materials were recently applied to the sensing of different types of biogenic metabolites, either ex vivo or in vivo. Even though the molecular gates methodology is mostly applied, there are some examples based on the covalent attachment of different dyes or fluorophores to the material. Thus, a way to profit the close arrangement of organic compounds decorating the MSN surface is to include two dyes, generating a microarray. Thus, Ding et al. [66], prepared a sensing material for biothiols with two fluorophores, 1,8-naphtyl-imide and rhodamine B derivatives, chemically bonded to silica mesoporous nanoparticles. The 2,4-dinitrobenzenesulfonyl group on the naphthalene group renders its fluorescence weak. When this group was removed, after a reaction with thiols, the naphthalene fluorescence appeared (Figure 26). By combining naphthalene and rhodamine fluorescence, changes in different extents in the presence of different biothiols resulted, allowing its discrimination. Moreover, changes in the response of the dual-fluorophore nanoparticle were observed on the modification of the concentration. A mini sensor array was thus generated as the analysis of the four signals provides a distinct recognition pattern, allowing the discrimination of four biothiols (H_2_S, Cys, Hcy, GSH) in aqueous solution and human serum.

Using terbium-bond mesoporous silica nanoparticles, M.D. Yilmaz et al., prepared a probe for the luminescent detection of the mycotoxin Ochratoxin A (OTA) [67].

**Figure 27:**
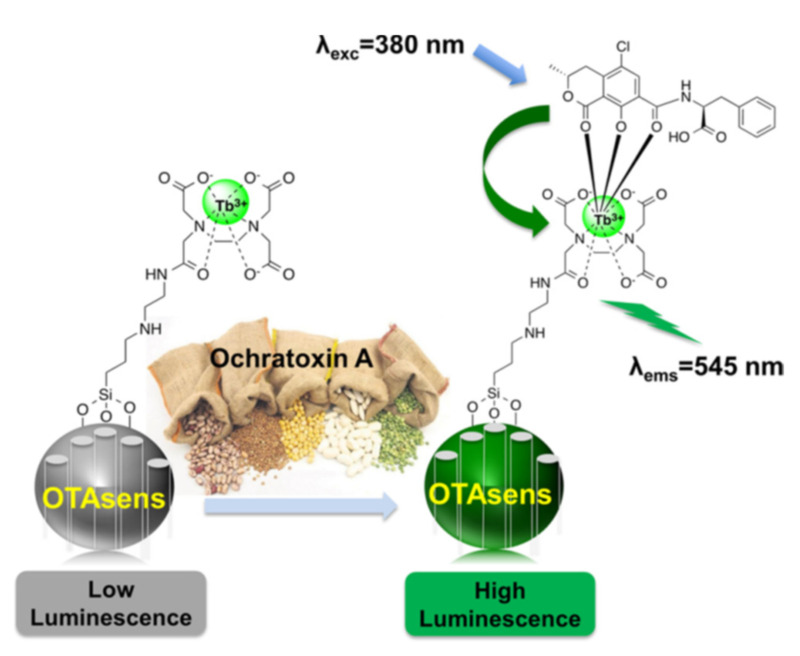
Detection of Ochratoxin A. Reprinted with permission from Osman Altunbas, Ayse Ozdas, M. Deniz Yilmaz (2020). Copyright 2020. Elsevier, Clearance Center.

Mesoporous silica nanoparticles EDTA functionalized were converted into the Tb^3+^-EDTA complex. The detection mechanism involved the binding of OTA to the Tb^3+^ cation. OTA acts as a sensitizer that transfers energy to the metal center, resulting in luminescence (antenna effect) (Figure 27). The limit of detection determined was 50 nM (20 ppb), and the probe was selective among other common mycotoxins and possible interfering compounds. Studies in real samples were carried out using OTA spiked fruit juices with average recoveries in the range from 80% to 93%.

**Figure 28:**
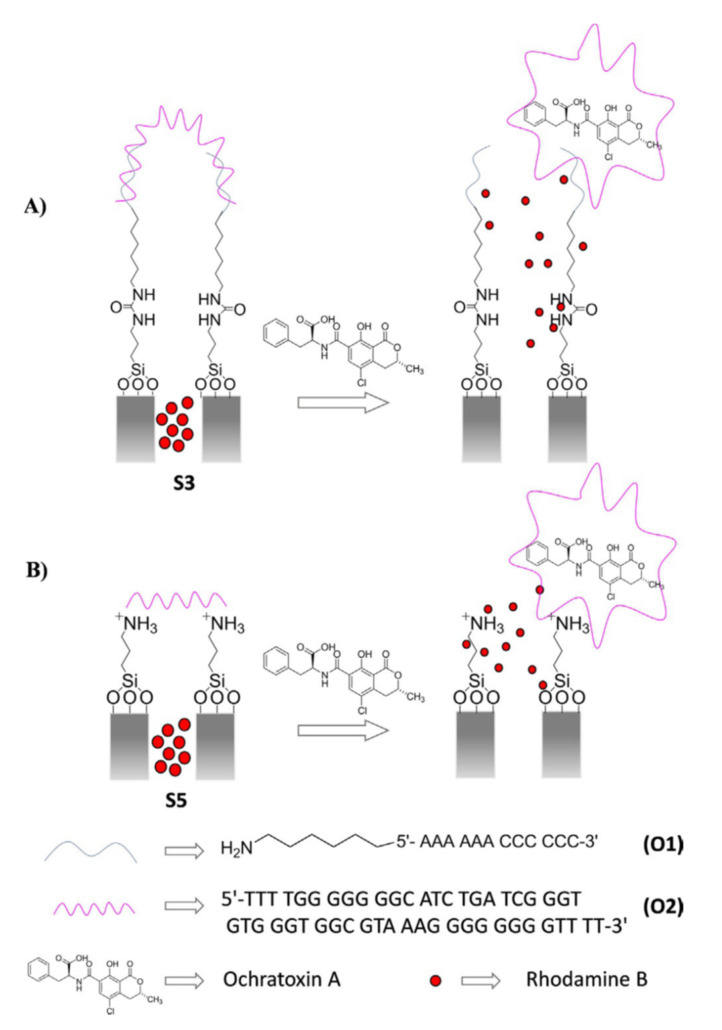
Detection of Ochratoxin A using aptamers (**A**) hybridized system (**B**) electrostatic bound approach. Reprinted with permission from Elena Aznar, Ramón Martínez-Máñez, Félix Sancenón, et al., (2017). Copyright 2017. John Wiley and Sons, Clearance Center.

Using mesoporous silica nanoparticles but following a different approach, R. Martínez-Máñez et al. reported two probes to detect the same mycotoxin (Ochratoxin A, OTA) [68]. In both cases, MSNs were loaded with rhodamine B and capped with an aptamer selective for OTA. Two different procedures for capped the nanoparticles were explored. In the first one, the external surface of the MSNs was functionalized with (3-isocyanatopropyl)triethoxysilane, and subsequently, a short DNA sequence, functionalized with an aminohexyl moiety at the 5′-end position, was covalently attached. To cap the pores, a single-stranded oligonucleotide which contains the specific sequence of the OTA aptamer, was hybridized. In the second approach, MSNs functionalized with (3-aminopropyl)triethoxysilane were partially charged at a neutral pH, and then the pores were capped with the charged aptamers through electrostatic interactions (Figure 28).

In the presence of OTA, the dye release was observed. The liberation was attributed to the displacement of the aptamer induced by the mycotoxin. Dye delivery, after the same time, was 60% in the electrostatic bound aptamer (with a limit of detection of 0.05 nM) and around 80% in the case of the hybridized system (with a limit of detection of 0.5 nM). Selectivity in relation to other mycotoxins such as aflatoxin B1 and fumonisin B1 was demonstrated. Studies with realistic wheat samples contaminated with OTA were carried out with positive results.

In a similar trend, Martínez-Máñez et al. [69] capped the pores of a MCM-41 loaded with rhodamine B with a bisphenol A aptamer (5′-TTT TGG GGG GCC GGT GGG TGG TCA GGT GGG ATA GCG TTC CGC GTA TGG CCC AGC GCA TCA CGG GTT CGC ACC AGG GGG GTT TT-3′) in such a way that the pores opened selectively only in the presence of Bisphenol-A, as its coordination with the aptamer displaced it. Concentrations as low as 3.5 µM could be detected. An amplification factor of 10 molecules of dye per molecule of BPA was determined.

Antibodies can also be used to cap the pores of MSMs; Rurack et al. [70] attached haptens, designed to couple with type-1 pyrethroid antibodies, to different architectures of MSMs. Best results were attained when the pore diameter matched with the antibody size. In the presence of pyrethroids, the antibody coupled with it and left the pore, allowing the release of the dye entrapped inside. Sensitivities down to the μg/kg range in less than 5 min, along with the strip arrangement nanodevice, allowed its application in point of need, such as the cabins of planes that spray pesticides.

In a simpler way, Costero et al. [71] generated a material with a double layer of a N-hydroxysuccinimide derivative that capped the pores of MCM-41 loaded with Rhodamine 6G. The interaction with biogenic amines spermine and spermidine dissolved the outer layer, liberating the dye. The detection was tested both in solution and in RAW 264.7 macrophages.

**Figure 29:**
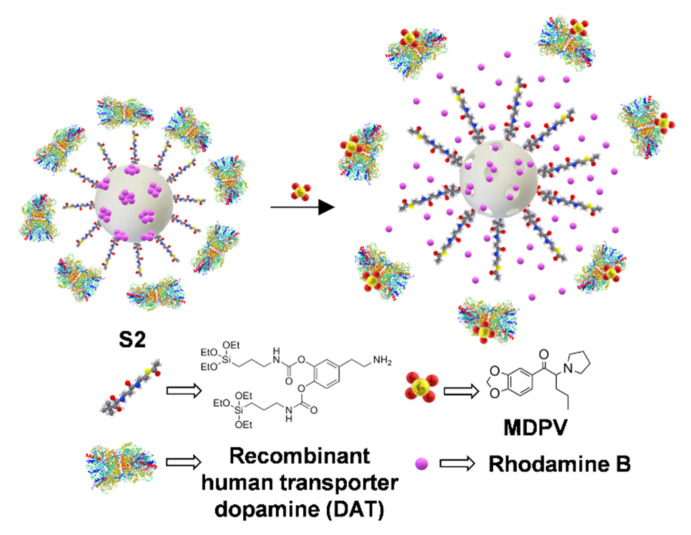
Sensing of Cannibal drug MDPV by displacement of recombinant human transporter dopamine.

In a similar way, other biomolecules able to interact with the analyte can be used for capping the pores. Sancenón et al. [72] prepared mesoporous silica nanoparticles functionalized with a dopamine derivative, and then, the recombinant human dopamine transporter (DAT) was added. As shown in Figure 29, its interaction with the dopamine moiety capped the pores, keeping the dye, rhodamine B, inside. In the presence of 3,4-methylenedioxypyrovalerone (MDPV), usually known as the Cannibal drug, DAT was displaced, and the liberated dye reported the drug with a detection limit of 5.2 µM. MDPV could be sensed in saliva and plasma, allowing its detection in site.

**Figure 30:**
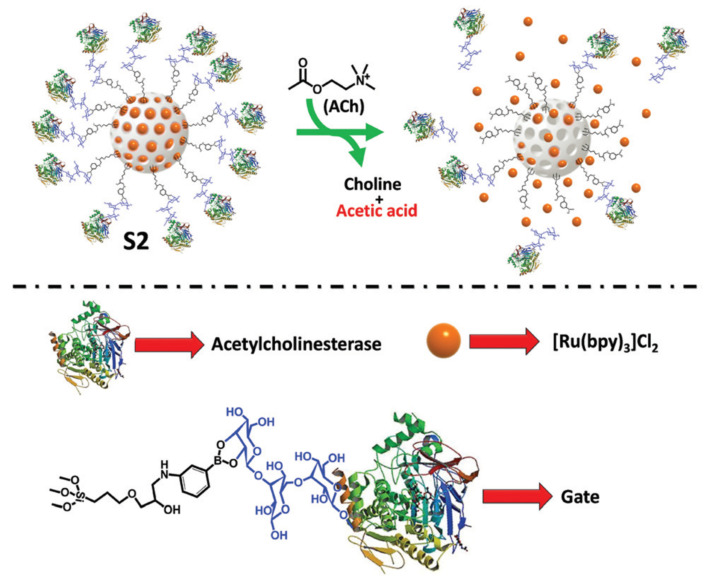
Representation of the design and performance of the acetylcholinesterase-capped nanodevice for cargo release in response to ACh.

The use of an enzyme for capping the pores, in addition to providing selectivity, allows for the use of the reaction it catalyzes to break any bond that opens the pores. This was elegantly demonstrated by Costero et al. [73] to sensing Acetylcholine selectively with MSN capped with acetylcholinesterase using boronic ester linkers (**S2**). On the reaction with Acetylcholine, choline was produced and acetic acid that hydrolyzed the boronic cyclic esters, releasing the enzyme and uncapping the pores, liberating the cargo (Figure 30). The material exhibits a similar behavior in complex biological environments such as HeLa cells.

### 2.6. Detection of Miscellaneous Compounds

**Figure 31:**
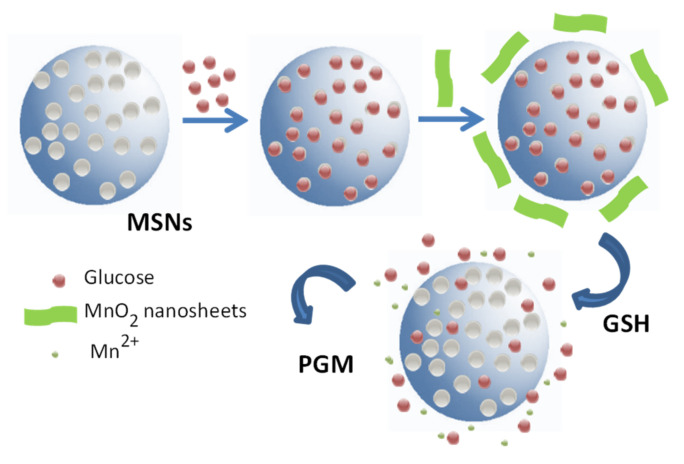
Sensing protocol based on MnO_2_ sheet reduction induced by GSH followed by glucose detection using a standard personal glucose meter (PGM).

Silica-based nanomaterials are also used in peptides and proteins’ detection. That, F. Qu et al. [74] reported the use of mesoporous silica nanoparticles gated with a MnO_2_ nanosheet for detecting glutation (GHS). Aminated mesoporous silica nanoparticles were loaded with glucose, and then the pores were caped with negatively charged MnO_2_ nanosheets through electrostatic interactions. The detection protocol was based on the reduction in the MnO_2_ sheet to Mn^2+^ induced by GHS. This reaction allows the glucose release that can be measured using a standard personal glucose meter (PGM) (Figure 31).

Different experimental parameters such as the concentration of glucose, concentration of MnO_2_ nanosheets and releasing time for glucose were optimized. Under the optimized conditions, a LOD for GSH of 34 nM was determined. The probe selectivity was evaluated in the presence of several potential interferents, including a wide range of electrolytes and biological species. Among the studied interferents, only ascorbic acid (AA) caused comparatively high PGM signals due to its red-ox characteristics. However, amino acids and electrolytes did not induce an obvious increase in the PGM signal. Even though AA can cause a response with the probe, its concentrations (μM levels) in biological samples are relatively lower than that of GSH. Studies with spiking human serum samples showed that the recovery of GSH ranged from 97.5% to 102.5%, and the RSD from 1.9% to 3.5%.

**Figure 32:**
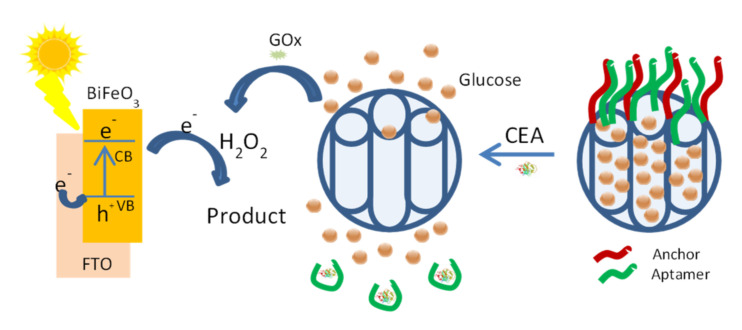
Schematic illustration of bismuth ferrite (BiFeO_3_)-based photoactive materials for the photoelectrochemical (PEC) detection of carcinoembryonic antigens (CEAs).

Following a related approach, M. Lu, D. Tang et al., reported a photoelectrochemical (PEC) sensing system for the detection of carcinoembryonic antigens (CEAs) [75]. The probe was based in the use of bismuth ferrite (BiFeO_3_) nanostructures as photoactive materials and capped mesoporous silica nanoparticles containing glucose inside the pores. Mesoporous silica nanoparticles that allow the amplification of the detectable signal were capped with a CEA aptamer. A competitive reaction between CEA and the immobilized anchor DNA on the MSNs for the partially hybridized aptamer opened the gate, and a large number of glucose molecules was released. The glucose in solution was oxidized by glucose oxidases (GOxs) to generate H_2_O_2_ that serves as the electron acceptor, which increases the photocurrent on the BiFeO_3_ photoactive material (Figure 32).

As well as the stability of the MSNs loaded with glucose and capped with the aptamer and the corresponding DNA strand, other parameters such as the amount of DNA using to cap the pores were evaluated. Under the optimized conditions, an estimated LOD of 1.5 pg mL^−1^ was determined. Selectivity was evaluated using other biomarkers such as alpha-fetoproteins (AFPs), prostate-specific antigens (PSAs), immunoglobulin G (IgG) and cancer antigen 125 (CA 125). The utility of the sensing system in real environments led to results comparable with these obtained by standard procedures.

**Figure 33:**
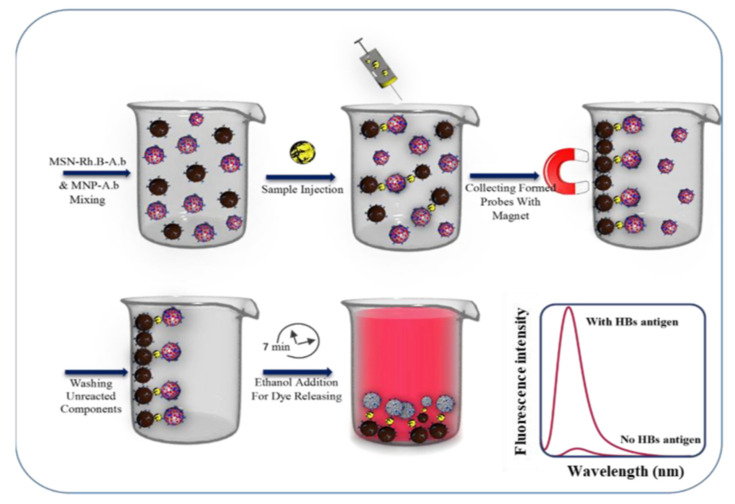
Representation of preparation steps and MSN-Rh. B/Ab-1-HBsAg- Ab-2/Fe_3_O_4_ MNP (sandwich probe) based immunoassay for HBsAg detection. Reprinted with permission from Zhaleh Ghafary, Rahman Hallaj, Abdollah salimi, Sudabeh Mafakheri (2021). Copyright 2021. Elsevier, Clearance Center.

R. Hallaj et al. reported a system based on magnetic mesoporous silica nanoparticles (MSNs) for detecting the hepatitis B virus surface antigen (HBV surface antigen) [76]. The authors took advantage of some characteristics of hybrid materials such as amplified detection, use of gated MSNs or MSNs with magnetic core. The proposed probe is formed by: (a) the Fe_3_O_4_ magnetic core nanoparticle (**Fe_3_O_4_ MNP@SiO_2_**) as a collector site and (b) the Rhodamine B loaded mesoporous silica nanoparticle (**MSN-Rh.B**) as a signal amplifier system. The later nanoparticles were modified with HBV antibodies (Ab) to form **MSN-Rh.B/Ab-1**. In a similar way, the magnetic nanoparticles’ surface was also functionalized with the corresponding antibody. When both nanoparticles were mixed, and analyte added, an interaction between both types of particles is produced. The magnetic properties of the sandwich complexes allowed for the concentration of the system using a magnetic field, and then the addition of ethanol induces the dye release that can be easily observed (Figure 33).

The limit of detection determined was the detection 9.95 ag/mL (with LOQ 29.9 ag/mL), and the precision of the probe using nine samples showed a relative standard deviation measured between the resulting fluorescence peaks by 6.0%. Several human serum samples spiked with different standard values of the HBsAg were evaluated with acceptable recoveries.

Modified mesoporous silica nanoparticles are also used to evaluate enzyme activity. In this field, Y.-B. Wang, H.-S. Wang et al., studied the deubiquitinating enzyme UCH-L1 activity through a Fluorescence Resonance Energy Transfer (FRET)-based nanoarchitecture [77]. Ubiquitination is a posttranslational modification of proteins that modulates their activity forming the corresponding isopeptides. This reaction takes place under the action of different enzymes, and it is a reversible process. Deubiquitinating enzymes (DUBs) catalyze the hydrolysis of isopeptide bonds giving rise to the regulation of the ubiquitination state of the target protein. DUBs play important roles in various diseases, and, for this reason, detection of their activity can be used in diagnostics and treatment of different pathological situations. It is well known that the fluorescence intensity of the lanthanide-based sensors can be affected by the coordination environment. In consequence, their confinement can affect their emissive characteristics. Mesoporous silica nanoparticles can control the confinement of lanthanides enhancing their fluorescent emission. Using this approach, the authors reported terbium (Tb)-loaded MSNs to be used to detect the activity of the DBU enzyme UCH-L1 as a model. The sensing protocol is based on a resonance energy transfer mechanism.

**Figure 34:**
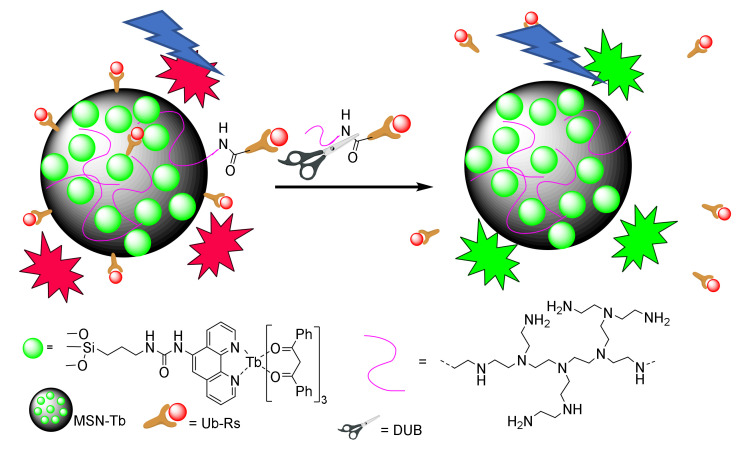
Activity evaluation of the enzyme UCH-L1.

Tb-complexes were bound to the pores giving rise to the modified material **MSN-Tb** where the emission properties of Tb are enhanced due to the confinement. On the other hand, amino groups of a polyamine compound were bound through an amide bond to the rhodamine B labelled Ubs (**Ub-Rs**). This modified amine compound was bound to the material giving rise to a system that, under light excitation, showed a FRET process from the Tb of the **MSN-Tb** to the rhodamine B moiety on the **Ub-Rs** (Figure 34). The main emission peak was mainly from the rhodamine B, while the emission of Tb was suppressed. In the presence of UCH-L1, the peptide bond was broken, inducing a break off of the FRET process. Due to this fact, the prepared probe could be used to evaluate the enzyme activity. The probe was selective to UCH-L1 in front of other compounds such as HSA, GSH, GOx, ATP and Cys. The lowest concentration of the enzyme detected using this probe was 5 nM.

**Figure 35:**
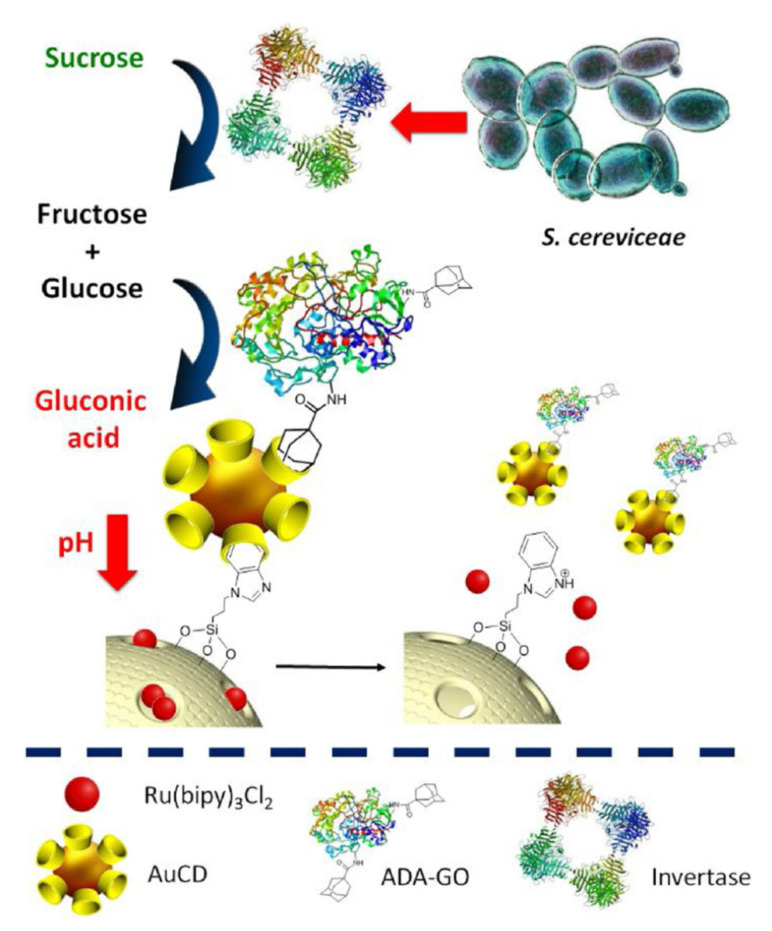
Sensing mechanism for Saccharomyces cerevisiae living cells detection. Reprinted with permission from Sandra Jimenez-Falcao, Anabel Villalonga, María Arévalo-Villena, Ana Briones-Pérez, Ramón Martínez-Máñez, Paloma Martínez-Ruiz, Reynaldo Villalonga (2020). Copyright 2020. Elsevier, Clearance Center.

The Martínez-Máñez research group developed a more elaborated system to detect Saccharomyces cerevisiae in living cells [78]. In this case, MSN loaded with tris(2,2′-bipyridyl)ruthenium(II) chloride hexahydrate ([Ru(bipy)_3_]Cl_2_) were capped with gold nanoparticles simultaneously functionalized with β-cyclodextrin and glucose oxidase (EC 1.1.3.4). The gold nanoparticles acted as a molecular gate through a supramolecular interaction between the β-cyclodextrin moiety and the benzoimidazol groups attached to the MSN. Saccharomyces cerevisiae living cells produced an extracellular invertase (EC 3.2.1.26) that transformed sucrose in fructose and glucose. The generated glucose was oxidized by the glucose oxidase bound to the gold nanoparticles. This oxidation generated gluconic acid that modified the pH protonating the benzoimidazol moiety and removing the cyclodextrin. All of this sequential process induced the dye release that could be observed and measured (Figure 35).

The limit of detection evaluated for the colorimetric sensor was 102 CFU/mL. Ten different sensing experiments toward 8·104 CFU/mL living S. cerevisiae cells were carried out, and a relative standard deviation of 2.7% was obtained. These data demonstrate the excellent reproducibility showed by the colorimetric sensors. In addition, the system was stable for at least two weeks at 4 °C. The response induced by other yeasts was less than 30% of that obtained for S. cerevisiae, probably due to the high capacity of this yeast to produce extracellular invertase. However, the response of the system could be affected by the presence of several sugars. This fact could be related to the capacity of S. cerevisiae cells to metabolize a large variety of carbohydrates as a carbon source. The nanosensor was validated in white wine samples by comparing the analytical results with those obtained by the conventional cell counting method with good agreement.

**Figure 36:**
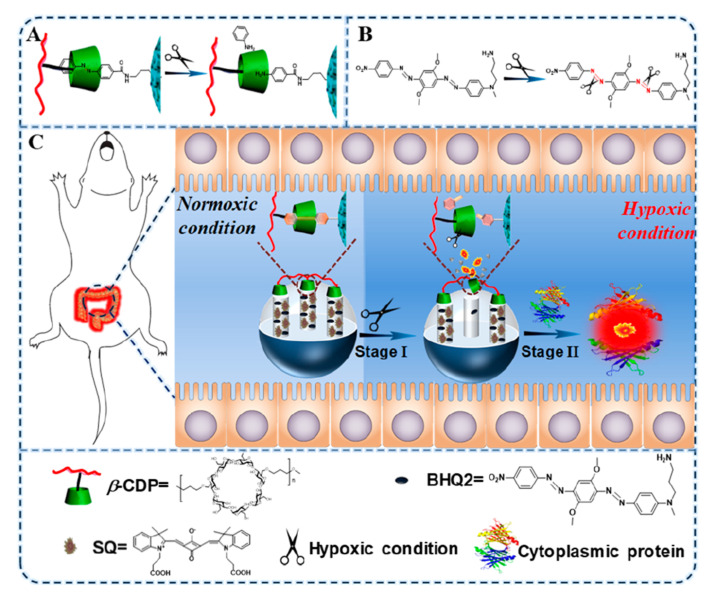
Protocol for detection of hypoxic environments using a fluorescent cascade amplification. (**A**) The response mechanism of azo/β-CDP and (**B**) the BHQ2 response to hypoxia. (**C**) Imaging of hypoxia associated with IBD by a cascade amplifier based on cytoplasmic protein-powered fluorescence cascade amplification. Reprinted with permission from Yibo Zhou, Sheng Yang, Jingru Guo, et al., (2020). Copyright 2013. ACS Publications.

Mesoporous silica nanoparticles are also used to identify different pathological environments. In this area, S. Yang, R. Yang et al., designed and evaluated a new probe to detect hypoxia associated with inflammatory bowel disease (IBD) [79]. There are many fluorescent probes described in the bibliography to detect the hypoxic environment, but usually they show a limited sensibility. These authors reported a signal amplification concept using a cytoplasmic protein as an amplification component, and an amplifying sensor with a high signal-to-background ratio. Following this strategy, they designed a fluorescence cascade amplifier, named **HCFA**, to image hypoxia associated with IBD in vivo. **HCFA** was constituted by mesoporous silica nanoparticles (MSN) functionalized with an azo-derivative and loaded with a mixture of squarylium dye (SQ) and black hole quencher 2 (BHQ2). The pores were capped using a β- cyclodextrin polymer (β-CDP) that reacted with the azo derivative through supramolecular interactions (Figure 36). Under hypoxic conditions, the azo group was transformed into the corresponding amino derivative, and this modification induced the separation of β-CDP with the concomitant release of the fluorescent dye. After this first step, SQ could bind with cytoplasmic proteins to enhance the fluorescence intensity for amplification of the signal.

The system remained in silence in normoxic conditions, but when the oxygen decreased to lower levels (<20%), the fluorescent signal appeared, and the intensity increased as the oxygen levels decreased. The probe was studied in HeLa cells, and it was not toxic at the studied concentrations. The high permeability of the probe across tissues allowed the system to be used to detect hypoxia induced by IBD in vivo.

## 3. Challenges and Perspectives

Materials based on MSNPs are of use in applications in different fields mainly in the environment and in health. Thus, pollution control and the purification of water, soil or the atmosphere can be accomplished using new materials able to detect and remove pollutant from different complex matrixes simultaneously. In this area, an important challenge is the preparation of both the recyclable system and materials to be used in a continuous way. In the health area, the possibility of applications is even higher. For example, detection of metabolytes or enzymes overexpressed in different pathological conditions seems to be a very promising research area to design easy protocols to be used in the early diagnosis of different diseases. For these applications, different type of materials should be prepared, able to increase the proportion of cargo and to guarantee its metabolization, avoiding the generation of toxic residues or its accumulation in organs. In this sense, a necessary improvement is related to the vectorization to direct the sensing materials to the therapeutic zone, minimizing possible negative side effects. The field of materials capped with molecular gates is another field of interest in both the detection and treatment of pathological situations. Even though many interesting advances were achieved in the last years, more specific systems must be prepared to increase the specificity of the gate opening, avoiding the cargo liberation in unwanted cells or tissues. Without any doubt, the area of theragnostic materials is one of the most promising because these systems cover two different objectives (diagnostic and treatment) simultaneously. In this sense, the use of MSNPs capped with molecular gates allows the release of active drugs from the material and simultaneously detects where the drug is being released. Going one step further, nanocontainers can be vectorized to facilitate their arrival to the point of action. Finally, the use of MSNPs containing metal atoms, or other in vivo contrast agents, in their structure can increase applicability by conferring the traceability of the materials along its route inside the body.

The examples included in the present review involve conventional observation techniques (naked-eye, UV-vis spectroscopy or fluorescence). However, other observation technologies have demonstrated to be of interest. In this sense, bulk waveguides (BWGs) can be applied in the porous material giving rise to new type of sensors. These light-guiding components can transduce the information about physical or chemical reactions happening inside the pores [80,81]. Another field to be explored is the use of MSNPs capped with molecular gates in the preparation of organic electrochemical transistor devices (OECTS). This application could be carried out in a simple way without more than loading the nanoparticles with some compound capable of interacting with the “gate” transistor. This approach will allow for the improvement not only in selectivity but also sensitivity, and presents a huge potential in health monitoring, as it condenses smart signal processing and neuromorphic computing by a single chip [82]. Similarly, the described materials could be also used to prepare amperometric sensors [83].

## 4. Conclusions 

The references included in this review demonstrated that there are many different analytes that can be detected using hybrid organic materials based on MSNPs. In some cases, the nanoparticles are only containers where the sensor is absorbed. In other systems, an organic molecule or a complex is covalently bound to the material. In some cases, the solid support can enhance selectivity or sensitivity whereas in others the inclusion of the sensor into the material prevents possible damages or photophysical modifications. The third approach considered in this review is the use of MSNPs capped with organic molecules with the function of molecular gates that are open in the presence of a specific stimulus that is the analyte presence. Different analytes can be detected using the described materials. Some of them are simple anions or cations whereas others are complex molecules such as endotoxins or even virous or enzymatic activity.

## Figures and Tables

**Table 1:** Synthetic conditions to obtain large pore silica nanoparticles (LPMSNs).

	SDS (g)	SDBS (g)	H_2_O mL	HCl (g)	TEO (mL)	APTES (mL)	T (°C)
**MSN1**	0.072	0	8.75	0.5	0.375	0.020	40
**MSN2**	0.072	0	8.75	0.5	0.375	0.040	40
**MSN3**	0.060	0.014	8.75	0.5	0.375	0.030	40
**MSN4**	0.060	0.014	8.75	0.5	0.375	0.040	40
**MSN5**	0.060	0.014	8.75	0.5	0.375	0.040	25

SDS sodium dodecyl sulphate, SDBS sodium dodecyl benzene sulfonate, TEOS Tetraethyl orthosilicate, APTES (3-Aminopropyl)triethoxysilane.

## References

[1] Aznar E., Oroval M., Pascual L., Murguía J.R., Martínez-Máñez R., Sancenón F. (2016). Gated Materials for On-Command Release of Guest Molecules. Chem. Rev..

[2] Sun Z., Cui G., Li H., Liu Y., Tian Y., Yan S. (2016). Multifunctional optical sensing probes based on organic—Inorganic hybrid composites. J. Mater. Chem. B.

[3] Alberti S., Soler-Illia G.J.A.A., Azzaroni O. (2015). Gated supramolecular chemistry in hybrid mesoporous silica nanoarchitectures: Controlled delivery and molecular transport in response to chemical, physical and biological stimuli. Chem. Commun..

[4] Visakh P.M., Martínez Morlanes M.J. (2016). Nanomaterials and Nanocomposites.

[5] Kresge C.T., Leonowicz M.E., Roth W.J., Vartuli J.C., Beck J.S. (1992). Ordered mesoporous molecular sieves synthesized by a liquid-crystal template mechanism. Nature.

[6] Pal N., Lee J.-H., Cho E.-B. (2020). Recent Trends in Morphology-Controlled Synthesis and Application of Mesoporous Silica Nanoparticles. Nanomaterials.

[7] Singh D., Saini R.K., Bhagwan S., Kurinec S.K. (2019). Emerging Photovoltaic Materials: Silicon and Beyond.

[8] Manj R.Z.A., Chen X., Rehman W.U., Zhu G., Luo W., Yang J. (2018). Big Potential from Silicon-Based Porous Nanomaterials: In Field of Energy Storage and Sensors. Front. Chem..

[9] Mehmood Y., Khan I.U., Shahzad Y., Khan R.U., Iqbal M.S., Khan H.A., Khalid I., Yousaf A.M., Khalid S.H., Asghar S. (2020). In-Vitro and In-Vivo Evaluation of Velpatasvir- Loaded Mesoporous Silica Scaffolds. A Prospective Carrier for Drug Bioavailability Enhancement. Pharmaceutics.

[10] Huang R., Shen Y.-W., Guan Y.-Y., Jiang Y.-X., Wu Y., Rahman K., Zhang L.-J., Liu H.-J., Luan X. (2020). Mesoporous silica nanoparticles: Facile surface functionalization and versatile biomedical applications in oncology. Acta Biomater..

[11] García-Fernández A., Aznar E., Martínez-Máñez R., Sancenón F. (2020). New Advances in In Vivo Applications of Gated Mesoporous Silica as Drug Delivery Nanocarriers. Small.

[12] Pontón I., Martí del Rio A., Gómez Gómez M., Sánchez-García D. (2020). Preparation and Applications of Organo-Silica Hybrid Mesoporous Silica Nanoparticles for the Co-Delivery of Drugs and Nucleic Acids. Nanomaterials.

[13] Chen L., Liu M., Zhou Q., Li X. (2020). Recent developments of mesoporous silica nanoparticles in biomedicine. Emergent Mater..

[14] Castillo R.R., Baeza A., Vallet-Regí M. (2017). Recent applications of the combination of mesoporous silica nanoparticles with nucleic acids: Development of bioresponsive devices, carriers and sensors. Biomater. Sci..

[15] Walcarius A. (2018). Silica-based electrochemical sensors and biosensors: Recent trends. Curr. Opin. Electrochem..

[16] Bagheri E., Ansari L., Abnous K., Taghdisi S., Seyed M., Ramezani P., Ramezani M., Alibolandi M. (2021). Silica–Quantum Dot Nanomaterials as a Versatile Sensing Platform. Crit. Rev. Anal. Chem..

[17] Gastaldi L., Ugazio E., Sapino S., Iliade P., Miletto I., Berlier G. (2012). Mesoporous silica as a carrier for topical application: The Trolox case study. Phys. Chem. Chem. Phys..

[18] Yang H., Zheng K., Zhang Z., Shi W., Jing S., Wang L., Zheng W., Zhao D., Xu J., Zhang P. (2012). Adsorption and protection of plasmid DNA on mesoporous silica nanoparticles modified with various amounts of organosilane. J. Colloid Interface Sci..

[19] Zimny K., Blin J.L., Stébé M.J. (2009). Ordered mesoporous silica templated by nonionic fluorinated liquid crystals. J. Phys. Chem. C.

[20] Wirnsberger G., Stucky G.D. (2000). Artificial Noses Sniff DNA. ChemPhysChem.

[21] Scott B.J., Wirnsberger G., Stucky G.D. (2001). Mesoporous and Mesostructured Materials for Optical Applications. Chem. Mater..

[22] Stein A., Melde B.J., Schroden R.C. (2000). Hybrid Inorganic–Organic Mesoporous Silicates—Nanoscopic Reactors Coming of Age. Adv. Mater..

[23] De Juan F., Ruiz-Hitzky E. (2000). Selective Functionalization of Mesoporous Silica. Adv. Mater..

[24] Lim M.H., Stein A. (1999). Comparative Studies of Grafting and Direct Syntheses of Inorganic−Organic Hybrid Mesoporous Materials. Chem. Mater..

[25] Climent E., Bernardos A., Martínez-Máñez R., Maquieira A., Marcos M.D., Pastor-Navarro N., Puchades R., Sancenón F., Soto J., Amorós P. (2009). Controlled Delivery Systems Using Antibody-Capped Mesoporous Nanocontainers. J. Am. Chem. Soc..

[26] Climent E., Martínez-Máñez R., Sancenón F., Marcos M.D., Soto J., Maquieira A., Amorós P. (2010). Controlled Delivery Using Oligonucleotide-Capped Mesoporous Silica Nanoparticles. Angew. Chem. Int. Ed..

[27] Coll C., Aznar E., Martínez-Máñez R., Marcos M.D., Sancenón F., Soto J., Amorós P., Cano J., Ruiz E. (2010). Fatty Acid Carboxylate- and Anionic Surfactant-Controlled Delivery Systems That Use Mesoporous Silica Supports. Chem. Eur. J..

[28] Candel I., Bernardos A., Climent E., Marcos M.D., Martínez-Máñez R., Sancenón F., Soto J., Costero A.M., Gil S., Parra M. (2011). Selective opening of nanoscopic capped mesoporous inorganic materials with nerve agent simulants; an application to design chromo-fluorogenic probes. Chem. Commun..

[29] Choudhury N., Saha B., De P. (2021). Recent progress in polymer-based optical chemosensors for Cu2+ and Hg2+ Ions: A comprehensive review. Eur. Polym. J..

[30] Ferreira Andrade G., Ferreir Soares D.C., Gouvêados Santos R., Martins Barros Sousa E. (2013). Mesoporous silica SBA-16 nanoparticles: Synthesis, physicochemical characterization, release profile, and in vitro cytocompatibility studies. Microporous Mesoporous Mater..

[31] Marcelo G.A., Mota J.P., Lodeiro C., Oliveira E. (2019). New dual colorimetric/fluorimetric probes for Hg^2+^ detection & extraction based on mesoporous SBA-16 nanoparticles containing porphyrin or rhodamine chromophores. Dye. Pigment..

[32] Chen F., Xiao F., Zhang W., Lin C., Wu Y. (2018). Highly Stable and NIR Luminescent Ru–LPMSN Hybrid Materials for Sensitive Detection of Cu^2+^ in Vivo. ACS Appl. Mater. Interfaces.

[33] Knežević N.Ž., Durand J.-O. (2015). Large pore mesoporous silica nanomaterials for application in delivery of biomolecules. Nanoscale.

[34] Kim H., Rao B.A., Jeong J., Angupillai S., Choi J.S., Nam J.-O., Lee C.-S., Son Y.-A. (2016). A rhodamine scaffold immobilized onto mesoporous silica as a fluorescent probe for the detection of Fe (III) and applications in bio-imaging and microfluidic chips. Sens. Actuators B Chem..

[35] Gai F., Zhou T., Chu G., Li Y., Liu Y., Huo Q., Akhtar F. (2016). Mixed anionic surfactant-templated mesoporous silica nanoparticles for fluorescence detection of Fe^3+^. Dalton Trans..

[36] Wu H., Jia J., Xu Y., Qian X., Zhu W. (2018). A reusable bifunctional fluorescent sensor for the detection and removal of silver ions in aqueous solutions. Sens. Actuators B Chem..

[37] Jimenez-Falcao S., Villalonga A., Parra-Nieto J., Llopis-Lorente A., Martinez-Ruiz P., Martínez-Máñez R., Villalonga R. (2020). Dithioacetal-mechanized mesoporous nanosensor for Hg(II) determination. Microporous Mesoporous Mater..

[38] Bell J., Climent E., Hecht M., Buurman M., Rurack K. (2016). Combining a Droplet-Based Microfluidic Tubing System with Gated Indicator Releasing Nanoparticles for Mercury Trace Detection. ACS Sens..

[39] Oroval M., Coll C., Bernardos A., Marcos M.D., Martínez-Mañez R., Shchukin D.G., Sancenón F. (2017). Selective Fluorogenic Sensing of As(III) Using Aptamer-Capped Nanomaterials. ACS Appl. Mater. Interfaces.

[40] Yu H., Chen C., Cao X., Liu Y., Zhou S., Wang P. (2017). Ratiometric fluorescent pH nanoprobes based on in situ assembling of fluorescence resonance energy transfer between fluorescent proteins. Anal. Bioanal. Chem..

[41] Cadenas E., Davies K.J.A. (2000). Mitochondrial free radical generation, oxidative stress, and aging. Free Rad. Biol. Med..

[42] Jha G., Roy S., Sahu P.K., Banerjee S., Anoop N., Rahaman A., Sarkar M. (2018). Free-radical sensing by using naphthalimide based mesoporous silica (MCM-41) nanoparticles: A combined fluorescence and cellular imaging study. Chem. Phys. Lett..

[43] Casula A., Llopis-Lorente A., Garau A., Isaia F., Kubicki M., Lippolis V., Sancenón F., Martínez-Máñez R., Owczarzak A., Santi C. (2017). A new class of silica-supported chromo-fluorogenic chemosensors for anion recognition based on a selenourea scaffold. Chem. Commun..

[44] Hussain R.A., Badshah A., Tahir M.N., Hassan T.U., Bano A. (2014). Synthesis, Chemical Characterization, DNA Binding, Antioxidant, Antibacterial, and Antifungal Activities of Ferrocence Incorporated Selenoureas. J. Biochem. Mol. Toxicol..

[45] Suzuki H., Iijima K., Moriya A., McElroy K., Scobie G., Fyfe V., McColl K.E.L. (2003). Conditions for acid catalysed luminal nitrosation are maximal at the gastric cardia. Gut.

[46] Taweekarn T., Wongniramaikul W., Limsakul W., Sriprom W., Phawachalotorn C., Choodum A. (2020). A novel colorimetric sensor based on modified mesoporous silica nanoparticles for rapid on-site detection of nitrite. Microchim. Acta.

[47] Ma Q., Lai Y., Gao J., Wang Q. (2016). Assay of fluoride by a novel organic–inorganic mesoporous nano-sized sensor. Luminescence.

[48] El Sayed S., Licchelli M., Martinez-Mañez R., Sancenon F. (2017). Capped Mesoporous Silica Nanoparticles for the Selective and Sensitive Detection of Cyanide. Chem. Asian J..

[49] Pasparakis M., Vandenabeele P. (2015). Necroptosis and its role in inflammation. Nature.

[50] Cheng D., Pan Y., Wang L., Zeng Z., Yuan L., Zhang X., Chang Y.-T. (2017). Selective Visualization of the Endogenous Peroxynitrite in an Inflamed Mouse Model by a Mitochondria-Targetable Two-Photon Ratiometric Fluorescent Probe. J. Am. Chem. Soc..

[51] Chen Z., Yan P., Zou L., Zhao M., Jiang J., Liu S., Zhang K.Y., Huang W., Zhao Q. (2018). Using Ultrafast Responsive Phosphorescent Nanoprobe to Visualize Elevated Peroxynitrite In Vitro and In Vivo via Ratiometric and Time-Resolved Photoluminescence Imaging. Adv. Healthc. Mater..

[52] Yang J., Cheng F., Zhu Z., Feng J., Xue M., Meng Z., Qiu L. (2020). An enhanced gas sensor based on SiO_2_@mesoporous MCM-41 core–shell nanocomposites for SO_2_ visual detection. Analyst.

[53] Wang L., Zhang H., Zhou X., Liu Y., Lei B. (2016). Preparation, characterization and oxygen sensing properties of luminescent carbon dots assembled mesoporous silica microspheres. J. Colloid Interface Sci..

[54] Yang Z., Wen J., Wang Q., Li Y., Zhao Y., Tian Y., Wang X., Cao X., Zhang Y., Lu G. (2019). Sensitive, Real-Time, and In-Vivo Oxygen Monitoring for Photodynamic Therapy by Multifunctional Mesoporous Nanosensors. ACS Appl. Mater. Interfaces.

[55] Kitajima N., Umehara Y., Son A., Kondo T., Tanabe K. (2018). Confinement of Singlet Oxygen Generated from Ruthenium Complex-Based Oxygen Sensor in the Pores of Mesoporous Silica Nanoparticles. Bioconjugate Chem..

[56] Gao Z., Qiao M., Tan M., Peng H., Ding L. (2020). Surface functionalization of mesoporous silica nanoparticles with pyronine derivative for selective detection of hydrogen sulfide in aqueous solution. Colloids Surf. A.

[57] El Sayed S., Pascual L., Licchelli M., Martínez-Mañez R., Gil S., Costero A.M., Sancenón F. (2016). Chromogenic Detection of Aqueous Formaldehyde Using Functionalized Silica Nanoparticles. ACS Appl. Mater. Interfaces.

[58] Climent E., Biyikal M., Gawlitza K., Dropa T., Urban M., Costero A.M., Martínez-Máñez R., Rurack K. (2017). Determination of the chemical warfare agents Sarin, Soman and Tabun in natural waters employing fluorescent hybrid silica materials. Sens. Actuators B.

[59] Climent E., Biyikal M., Gawlitza K., Dropa T., Urban M., Costero A.M., Martínez-Máñez R., Rurack K. (2016). A Rapid and Sensitive Strip-Based Quick Test for Nerve Agents Tabun, Sarin, and Soman Using BODIPY-Modified Silica Materials. Chem. Eur. J..

[60] Pascual L., El Sayed S., Martínez-Mañez R., Costero A.M., Gil S., Gaviña P., Sancenón F. (2016). Acetylcholinesterase-Capped Mesoporous Silica Nanoparticles That Open in the Presence of Diisopropylfluorophosphate (a Sarin or Soman Simulant). Org. Lett..

[61] Juárez L.A., Costero A.M., Parra M., Gaviña P., Gil S., Martínez-Máñez R., Sancenón F. (2017). NO_2_-controlled cargo delivery from gated silica mesoporous nanoparticles. Chem. Commun..

[62] Wang C., Li Q., Wang B., Li D., Yu J. (2018). Fluorescent sensors based on AIEgen-functionalised mesoporous silica nanoparticles for the detection of explosives and antibiotics. Inorg. Chem. Front..

[63] Kim Y., Min Lee K., Young Chang J. (2017). Highly luminescent tetra(biphenyl-4-yl)ethene-grafted molecularly imprinted mesoporous silica nanoparticles for fluorescent sensing of diethylstilbestrol. Sens. Actuators B.

[64] Tan S.Y., Teh C., Yen Ang C., Li M., Li P., Korzh V., Zhao Y. (2017). Responsive mesoporous silica nanoparticles for sensing of hydrogen peroxide and simultaneous treatment toward heart failure. Nanoscale.

[65] Liu C., Chen W., Qing Z., Zheng J., Xiao Y., Yang S., Wang L., Li Y., Yang R. (2016). In Vivo Lighted Fluorescence via Fenton Reaction: Approach for Imaging of Hydrogen Peroxide in Living Systems. Anal. Chem..

[66] Gao Z., Wang Z., Qiao M., Peng H., Ding L., Fang Y. (2020). Mesoporous silica nanoparticles-based fluorescent mini sensor array with dual emission for discrimination of biothiols. Colloids Surf. A.

[67] Altunbasa O., Ozdasa A., Yilmaz M.D. (2020). Luminescent detection of Ochratoxin A using terbium chelated mesoporous silica nanoparticles. J. Hazard. Mater..

[68] Ribes A., Santiago-Felipe S., Bernardos A., Marcos M.D., Pardo T., Sancenón F., Martínez-Mañez R., Aznar E. (2017). Two New Fluorogenic Aptasensors Based on Capped Mesoporous Silica Nanoparticles to Detect Ochratoxin A. ChemistryOpen.

[69] Ribes A., Aznar E., Bernardos A., Marcos M.D., Amorós P., Martínez-Máñez R., Sancenón F. (2017). Fluorogenic Sensing of Carcinogenic Bisphenol A using Aptamer-Capped Mesoporous Silica Nanoparticles. Chem. Eur. J..

[70] Costa E., Climent E., Gawlitza K., Wan W., Weller M.G., Rurack K. (2020). Optimization of analytical assay performance of antibody-gated indicator-releasing mesoporous silica particles. J. Mater. Chem. B.

[71] Barros M., López-Carrasco A., Amorós P., Gil S., Gaviña P., Parra M., El Haskouri J., Terencio M.C., Costero A.M. (2021). Chromogenic Chemodosimeter Based on Capped Silica Particles to Detect Spermine and Spermidine. Nanomaterials.

[72] Garrido E., Alfonso M., Díaz de Greñu B., Marcos M.D., Costero A.M., Gil S., Sancenón F., Martínez-Mañez R. (2020). A Sensitive Nanosensor for the In Situ Detection of the Cannibal Drug. ACS Sens..

[73] Godoy-Reyes T.M., Llopis-Lorente A., García-Fernández A., Gaviña P., Costero A.M., Martínez-Máñez R., Sancenón F. (2019). Acetylcholine-responsive cargo release using acetylcholinesterase-capped nanomaterials. Chem. Commun..

[74] Tan Q., Zhang R., Kong R., Kong W., Zhao W., Qu F. (2018). Detection of glutathione based on MnO_2_ nanosheet-gated mesoporous silica nanoparticles and target induced release of glucose measured with a portable glucose meter. Microchim. Acta.

[75] Zhou Q., Lin Y., Lu M., Tang D. (2017). Bismuth ferrite-based photoactive materials for the photoelectrochemical detection of disease biomarkers coupled with multifunctional mesoporous silica nanoparticles. J. Mater. Chem. B.

[76] Ghafary Z., Hallaj R., Salimi A., Mafakheri S. (2021). Ultrasensitive fluorescence immunosensor based on mesoporous silica and magnetic nanoparticles: Capture and release strategy. Spectrochim. Acta Part. A Mol. Biomol. Spectrosc..

[77] Liang Y.-Y., Zhang J., Cui H., Shao Z.-S., Cheng C., Wang Y.-B., Wang H.-S. (2020). Fluorescence resonance energy transfer (FRET)-based nanoarchitecture for monitoring deubiquitinating enzyme activity. Chem. Commun..

[78] Jimenez-Falcao S., Villalonga A., Arévalo-Villena M., Briones-Pérez A., Martínez-Máñez R., Martínez-Ruiz P., Villalonga R. (2020). Enzyme-controlled mesoporous nanosensor for the detection of living *Saccharomyces cerevisiae*. Sens. Actuators B Chem..

[79] Zhou Y., Yang S., Guo J., Dong H., Yin K., Huang W.T., Yang R. (2020). In Vivo Imaging of Hypoxia Associated with Inflammatory Bowel Disease by a Cytoplasmic Protein-Powered Fluorescence Cascade Amplifier. Anal. Chem..

[80] Veiko V.P., Zakoldaev R.A., Sergeev M.M., Pavel A., Danilov P.A., Kudryashov S.I., Kostiuk G.K., Sivers A.N., Ionin A.A., Antropova T.V. (2018). Direct laser writing of barriers with controllable permeability in porous glass. Opt. Express.

[81] Lijing Z., Zakoldaev R.A., Sergeev M.M., Veiko V.P. (2020). Fluorescent Bulk Waveguide Sensor in Porous Glass: Concept, Fabrication, and Testing. Nanomaterials.

[82] Shen H., Abtahi A., Lussem B., Boudouris B.W., Mei J. (2021). Device Engineering in Organic Electrochemical Transistors toward Multifunctional Applications. ACS Appl. Electron. Mater..

[83] Hwang C., Park N., Kim E.S., Kim M., Kim S.D., Park S., Kim N.Y., Kim J.H. (2021). Ultra-fast and recyclable DNA biosensor for point-of-care detection of SARS-CoV-2 (COVID-19). Biosens. Bioelectron..

